# Mesp1 Marked Cardiac Progenitor Cells Repair Infarcted Mouse Hearts

**DOI:** 10.1038/srep31457

**Published:** 2016-08-19

**Authors:** Yu Liu, Li Chen, Andrea Diaz Diaz, Ashley Benham, Xueping Xu, Cori S. Wijaya, Faisal Fa’ak, Weijia Luo, Benjamin Soibam, Alon Azares, Wei Yu, Qiongying Lyu, M. David Stewart, Preethi Gunaratne, Austin Cooney, Bradley K. McConnell, Robert J. Schwartz

**Affiliations:** 1 Department of Biology and Biochemistry, University of Houston, Houston, TX 77204, USA; 2 Department of Pharmacological and Pharmaceutical Sciences, University of Houston, Houston, TX 77204, USA; 3 Department of Molecular and Cellular Biology, Baylor College of Medicine, Houston, TX 77030, USA; 4 Stem Cell Engineering, Texas Heart Institute at St. Luke’s Episcopal Hospital, Houston, TX 77030, USA.; 5 Department of Computer Science and Engineering Technology, University of Houston-Downtown, Houston, 77002, USA.

## Abstract

Mesp1 directs multipotential cardiovascular cell fates, even though it’s transiently induced prior to the appearance of the cardiac progenitor program. Tracing Mesp1-expressing cells and their progeny allows isolation and characterization of the earliest cardiovascular progenitor cells. Studying the biology of Mesp1-CPCs in cell culture and ischemic disease models is an important initial step toward using them for heart disease treatment. Because of Mesp1’s transitory nature, Mesp1-CPC lineages were traced by following EYFP expression in murine Mesp1^Cre/+^; Rosa26^EYFP/+^ ES cells. We captured EYFP+ cells that strongly expressed cardiac mesoderm markers and cardiac transcription factors, but not pluripotent or nascent mesoderm markers. BMP2/4 treatment led to the expansion of EYFP+ cells, while Wnt3a and Activin were marginally effective. BMP2/4 exposure readily led EYFP+ cells to endothelial and smooth muscle cells, but inhibition of the canonical Wnt signaling was required to enter the cardiomyocyte fate. Injected mouse pre-contractile Mesp1-EYFP+ CPCs improved the survivability of injured mice and restored the functional performance of infarcted hearts for at least 3 months. Mesp1-EYFP+ cells are *bona fide* CPCs and they integrated well in infarcted hearts and emerged *de novo* into terminally differentiated cardiac myocytes, smooth muscle and vascular endothelial cells.

Heart failure is one of the leading causes of death globally. It’s caused by acute and/or chronic loss of cardiac myocytes in the human heart, which lacks sufficient regenerative capacity. Unfortunately, heart transplants are the only means to cure terminal stage heart failure limited by the availability of donor hearts. A great deal of effort has been placed into cell-based therapy, hoping that exogenously delivered cells could replace injured cardiomyocytes (CMs) and restore pump function. However, efforts to date have only led to a limited degree of success. Newer cutting-edge regimens used cardiac transcription factors Gata4, Mef2c; Tbx5 (or GMT plus Hand2) to reprogram cardiac fibroblasts *in vivo*, and observed decreased scar formation and improved pump function post myocardium infarction^1^,^2^,^3^. Despite their perceived benefits, cell regenerative efficiency was low and the mechanism behind the improved outcome was not clear. We felt that the use of cardiac progenitor cells (CPCs) represent a legitimate alternative, because they offer the advantage of multipotency and higher proliferation over terminally differentiated cells. CPCs can further differentiate into more than just CMs and hence have the potential to achieve concerted therapeutic effect.

Thus far two major cardiac progenitor populations were defined. The first population was delineated by the appearance of two cell surface markers, Flk1/KDR and PDGFRa in differentiating ES cells. This population was widely used as a means to enrich cardiac progenitors^4^,^5^,^6^. Growth factor supplements that trigger the expression of the two markers greatly increased the yield of cardiac myocytes. Both markers have relatively broad expression in extra embryonic tissues and early embryos, which overlap but lack a specific role in cardiac myogenesis. The loss of Flk1 caused developmental defects primarily in hematopoietic and endothelial lineages, while the loss of PDGFRa resulted in somatic patterning defects and neural crest-originated defects^7^,^8^. Accordingly, Flk1/KDR and PDGFRa double positive cells represent an early mesoderm population enriched for cardiac mesoderm cells. A second population was defined by the appearance of cardiac transcription factors, such as Nkx2-5+, which marked cardiomyocyte and vascular smooth muscle progeny, but not endothelial cells^9^. Yet, another was marked by Isl1+, which contributed to the three major cardiac lineages, but was limited to the second heart field^10^. We considered a more likely mesoderm subpopulation, in which transient Mesp1 expression is upstream of Nkx2.5 and Isl1 and generates the CPCs that give rise to the cardiovascular system.

Mesp1 is expressed at the onset of gastrulation, along the primitive streak and in the premesoderm that eventually gives rise to the heart. Lineage tracing studies using Cre recombinase knocked into the Mesp1 locus revealed that Mesp1-expressing cells mainly contributed to the mesoderm component of the amnion, and to the myocardium of the heart tube^11^,^12^,^13^. Homologous disruption of the Mesp1 gene resulted in aberrant cardiac morphogenesis. Mesp1-expressing cells were delayed in migrating from the primitive streak to the heart field, in the homozygous Mesp1-deficient embryos^12^,^13^. Furthermore, simultaneous disruption of both Mesp1 and Mesp2 genes (dKO) led to the complete loss of posterior structures including heart, somites and gut. Chimera analysis, however, showed that Mesp1 and Mesp2 dKO cells still contributed to the formation of somites and gut, but not heart^14^. These data indicate that Mesp1 and Mesp2 are essential for the formation of the cardiac lineages.

Mesp1 drives ES cells toward the cardiac fate. Transient expression of Mesp1 accelerated and enhanced the appearance of cardiac progenitors^15^,^16^,^17^. Several transcription factors with known cardiac function, including Hand2, Gata4, myocardin, FoxH1, FoxC1 were upregulated within 18 hours following Mesp1 induction. Mesp1 also binds to the promoter region of several core cardiac transcription factors, including Hand2, Nkx2-5, Myocardin and Gata4^15^. These findings suggest that Mesp1 sits on the top of the gene regulatory network for cardiogenesis. Interestingly, Mesp1 directly regulates Dkk-1, which blocks canonical Wnt signaling, an established mechanism of heart induction^16^.

Despite the central role of Mesp1 in cardiogenesis, its expression in the developing embryo is brief. Mesp1 appears at the onset of mesoderm formation (E6.0) and virtually disappears before the emergence of the cardiac crescent (E7.0); thus, Mesp1 likely imparts important regulatory information; albeit transiently, that may be required for later stages of cardiac development^11^,^12^,^13^. Here, we use Mesp1Cre to trace the Mesp1 lineage in murine ES cells. The Mesp1-CPCs contribute to the repair of post-MI hearts by supplying cardiac myocytes, vascular smooth muscle and endothelial cells, accompanied by significant functional improvement.

## Materials and Methods

### Generation of embryonic stem cell lines and cell culture

The Mesp1-Cre mouse strain was developed by Yumiko Saga^13^. The Rosa26-EYFP mouse strain was developed by Frank Costantini^18^. We had crossed these two strains to create the Mesp1^Cre/+^; Rosa26^EYFP/+^ ESC reporter line, as we reported in Soibam *et al*.^19^. ES cell lines were maintained in ES cell media [Dulbecco's modified Eagle's medium (high glucose; Gibco, Gaithersburg, MD, USA) supplemented with 15% fetal bovine serum (FBS; HyClone, Logan, UT, USA), 1 mM sodium pyruvate, 1% MEM nonessential amino acids, 1 × 10^3^ U/ml murine leukemia inhibitory factor (ESGRO-LIF; Gibco), 0.1 mM ß-mercaptoethanol, 50 U/ml penicillin, and 50 μg/ml streptomycin].

ES cell differentiation was performed under serum-containing or serum-free conditions. Serum-containing differentiation media (SCDM) was similar to ES cell media except that SCDM contained 20% FBS and no LIF. To induce EB formation and differentiation in SCDM, the cells were grown as 20 μl hanging droplets (2 × 10^4^ cells/ml) for 5 consecutive days^20^. EBs were collected on day 5, and plated on 0.1% gelatin-coated dishes. The medium was replaced the next day and every two days thereafter. Serum-free differentiation condition was used exclusively whenever growth factors were supplemented during the procedures. If not otherwise indicated, the cells were grown as 20 ul hanging droplets (2 × 10^4^ cells/ml) for 2 days in serum-free differentiation media (SFDM, a 3:1 mixture of IMDM/F12 containing 0.05% BSA, 100 U/ml penicillin G, 100 μg/ml streptomycin sulfate, 2 mM L-glutamate, 1x chemically defined lipids, 450 uM 1-thioglycerol, 0.5x B27, 1X N2, 0.4mg/ml PVA, 10 ug/ml Insulin, 1 uM Y-27632 and 20 ng/ml BMP4). EBs were transferred into an ultra-low attachment 6-well plate and cultured in SFDM omitting PVA and Insulin for 24 hours. Next, EBs were dissociated by 0.25% Trypsin and plated to a collagen I-precoated plate. Typically, beating clusters were observed 6 days after the initiation of hanging droplets.

The following growth factors, inhibitors and small molecules were used as cell culture supplements when indicated: Activin (R & D Systems, 25 ng/ml), BMP2 (R & D Systems, 20 ng/ml), BMP4 (R & D Systems, 20 ng/ml), DLL1 (R & D Systems, 100 ng/ml), bFGF (R & D Systems, 10 ng/ml), FGF8 (R & D Systems, 10 ng/ml), Tgfb (R & D Systems, 20 ng/ml), Wnt3a (R & D Systems, 100 ng/ml), IWR1 (Tocris, 5 μM), SB431542 (Tocris, 10 μM).

### FACS analysis and sorting

EBs or cells cultured as monolayers, were first washed with PBS, and dissociated in 0.25% Trypsin. Next, ES cell media was used to neutralize the reaction and cells were collected by centrifuge, 1000 rpm for 5 minutes. The cells were then suspended in 1-2 ml of ES media, passed through a cell strainer and into a conical FACS tube. FACS analysis was performed on a BD LSRII flow cytometer (BD Biosciences). Sorting was performed on a FACSaria II flowcytometer (BD Biosciences) and FACS data was processed with Flowjo software (Tree Star).

For staining of Flk1, cells were incubated with primary antibodies (Flk1, 1:100) diluted in 5% BSA/PBS for 30 minutes on ice. Next, the cells were washed with 5% BSA/PBS, and incubated with secondary antibodies (APC-conjugated) diluted in 5% BSA/PBS. For staining of PDGFRa, PE-conjugated anti-PDGFRa (1:100) was used.

### Real-time quantitative RT-PCR

RNA (100 ng) was subjected to quantitative RT-PCR using the Taqman One-Step RT-PCR Master Mix reagent (Applied Biosystems) and a 7900HT Fast Real-time PCR System (Applied Biosystems). Copy number for each transcript is expressed relative to that of glyceraldehyde-3-phosphate dehydrogenase (GAPDH), as a constitutive control. Sequences of primer sets and probes are provided in Supplemental Table 1.

### Whole genome microarray

RNA was isolated using TRIzol (Invitrogen) and RNeasy reagents (Qiagen). The integrity and concentration of RNA samples was determined using RNA 6000 Nano LabChip kits and an Agilent 2100 Bioanalyzer. Whole genome microarray analysis was performed on OneArray Mouse Whole Genome Array (Phalanx Biotech Group). The raw data were re-scaled to account for the differences in individual hybridization intensities. The heatmap represents relative expression as z-scores. The z-score of a gene was computed by using the expressions of all the genes both in EYFP+ and EYFP- cells. Gene Ontology analysis was performed on genes falling within the condition, p < 0.01, fold change >2, using DAVID (http://david.abcc.ncifcrf.gov). GO network analysis was performed using enrichment Map (http://baderlab.org/Software/EnrichmentMap) with p < 0.01 as the cutoff.

### Immunostaining and fluorescent microscopy

See Supplemental Methods.

### Myocardial infarction surgery and cell delivery

University of Houston IACUC and Baylor College of Medicine IACUC approved all animal protocols. An animal model of ischemic myocardial infarction (MI) in 8–10 week old adult male C.B-17 SCID mice was induced by chronic (permanent) ligation of the LAD artery^21^ and as we have similarly performed in rats^22^. A single experienced animal microsurgery technician (ADD) performed all the procedure. Ligation was verified by immediate myocardial blanching and anterior wall dysfunction. Animals were randomized into the various groups: (1) sham-operated, (2) MI + PBS, (3) MI + Mesp1-CPCs, and (4) un-operated-controls. CPCs were injected using 5 (2 μl) injections (total volume = 10 μl) each with 20,000 CPCs (total number of cells  = 0.1 × 10^6^ cells) into the infarct and border zone areas, immediately following LAD artery ligation.

### Cardiac magnetic resonance imaging and transthoracic echocardiography

See Supplemental methods.

### Statistical analysis

Data were processed using Microsoft Excel and GraphPad Prism 5.0. All values are expressed, as the mean ± S.E.M. Comparisons between two groups were determined using unpaired 2-tailed Student’s *t* test. Analysis was performed using one-way ANOVA, followed by a Tukey’s *post hoc* multiple comparison test when multiple groups were compared. Kaplan-Meier survival analysis was performed using the Log-rank (Mantel-Cox) test. *P* values less than 0.05 were considered significant.

### Ethical approvals and informed consent

All experiments were performed in accordance with approved guidelines and regulations. Furthermore, all animal studies have been approved by the Institutional Animal Care and Use Committee (IACUC) and ethics committee at the University of Houston (UH; #UH-ACP-13-022) and the Baylor College of Medicine (BCM; #BCM-AN-5199). Animal care was provided in Association for Assessment and Accreditation of Laboratory Animal Care (AAALAC) accredited animal barrier facilities at UH and BCM located within the Texas Medical Center (TMC) and have therefore been performed in accordance with the ethical standards laid down in the 1964 Declaration of Helsinki and its later amendments. Also, all authors of this report gave their informed consent prior to their inclusion in the study.

## Results

### Mesp1-EYFP+ lineage tracks cardiac progenitor cells

**Figure 1:**
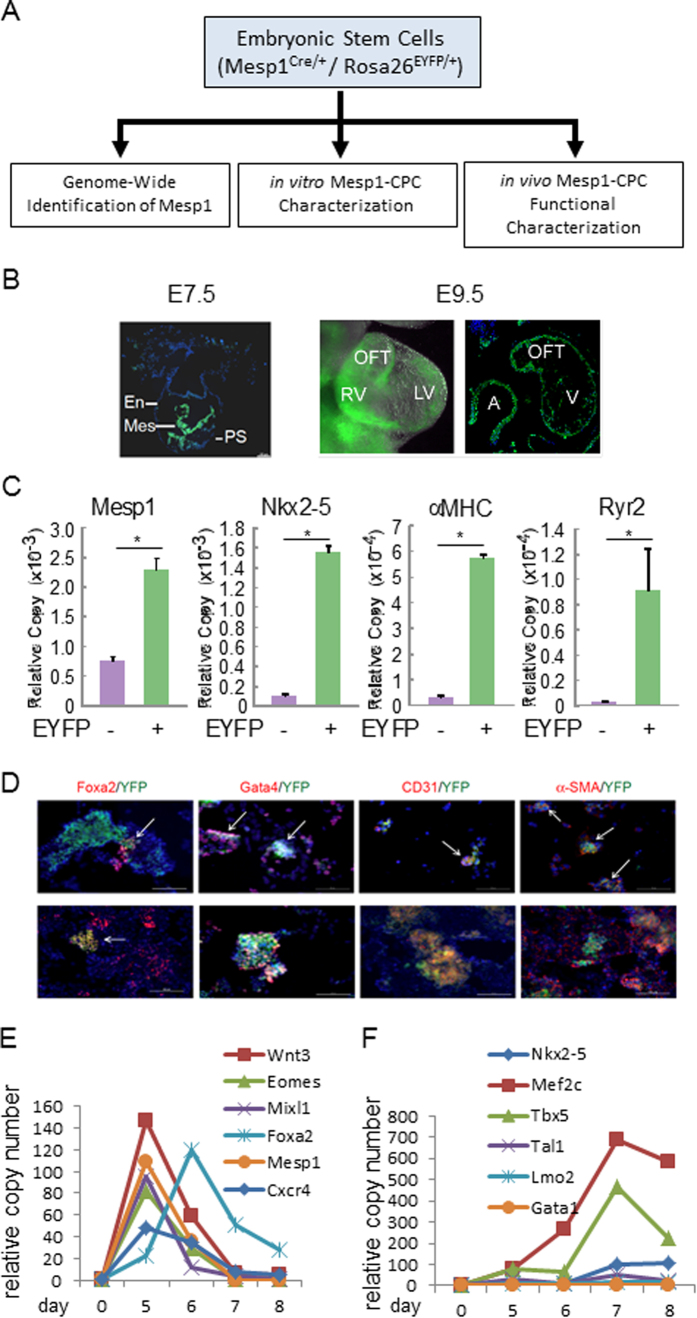
Mesp1-lineage cells are a CPC-enriched population, which contain endoderm components. (**A**) Schematic characterization of embryonic stem cells (Mesp1^Cre/+^/Rosa26^EYFP/+^) in this study; *in vitro* Mesp1-CPC characterization and *in vivo* Mesp1-CPC functional characterization. The genome-wide identification of Mesp1 targets, and the establishment of the reporter ES cell line, was previously published^19^. (**B**) Mesp1-EYFP+ signals were located in the mesoderm in E7.5 and E9.5 embryos. E7.5 panel:an embryonic sagettal section showing EYFP signal mainly in the mesoderm (Mes), and less in endoderm (En) and primitive streak (PS). E9.5 panel(left): an embryonic heart showing EYFP signal in left ventricle (LV), right ventricle (RV) and out flow tract (OFT). E9.5 panel (right): an embryonic heart section showing EYFP signal in atrium (A), ventricle (V) and out flow tract (OFT). (**C**) Enrichment of cardiac-related genes in the EYFP+ population. Gene expression levels were measured by real-time RT-PCR. Mesp1 was measured at day 5 of EB culture. Nkx2-5, αMHC and Ryr2 were measured at day 8 of EB culture. N ≥ 3; *p < 0.05 versus control cells. (**D**) Mesp1-lineage cells are a CPC-enriched population, which contain endoderm components. Co-localization of EYFP and lineage markers in differentiating ES cells. Day 5 EBs were stained by EYFP, Foxa2, Gata4, CD31, and α-SMA antibodies. (**E**,**F**) Expression of cardiac and hematopoietic transcription factors in EYFP+ cells during differentiation. (**E**) Transcription factors Wnt3, Eomes, Mixl1, Foxa2, Mesp1 and Cxcr4 showed strong induction between days 4–6. (**F**) Cardiac transcription factors Nkx2-5, Mef2c and Tbx5 showed much stronger induction over the time course than hematopoietic transcription factors Tal1, Lmo2 and Gata1.

To track the Mesp1-marked progenitor cell lineage, we previously crossed the murine Mesp1^Cre/+^ line with the Rosa26^EYFP/EYFP^ line to generate a Mesp1^Cre/+^; Rosa26^EYFP/+^ ESC reporter line as we reported in Solbam *et al*.^19^. We found that MESP1 primarily drove the formation of mesendoderm, a common ancestor of mesoderm and endoderm properties. Here, to characterize further specification of these Mesp1-EYFP+ cells, we investigated their contribution to multiple lineages in ES cell differentiation (*in vitro* characterization) and heart disease (*in vivo* characterization) (Fig. 1A). In developing embryos, majority of EYFP+ signals were first located in the mesoderm, and subsequently in the heart (Fig. 1B). In a standard serum-containing embryoid body culture protocol, Mesp1 transcripts were significantly enriched in the EYFP+ fraction. Though Mesp1 transcripts were also present in the EYFP- fraction, this likely reflects the delay between activation of the Mesp1 locus (Cre) and subsequent Cre-mediated activation of the Rosa locus (EYFP) in EYFP- cells, which would later turn EYFP+. At day 8, Nkx2.5, αMHC, and Ryr2 transcripts were almost exclusively present in EYFP+ cells, supporting that cardiomyocytes arise from Mesp1+ progenitors (Fig. 1C).

Others and we recently documented that the Mesp1-lineage cells were a CPC-enriched population, which also contained endodermal and hematopoietic components. Consistently, EYFP+ signal colocalized with endoderm factors Foxa2 and Gata4 in day 4 differentiated ES cells (Fig. 1D). However, enrichment of cardiac-related genes, including Nkx2-5, Tbx5 and Mef2c, in the EYFP+ population were much greater than hematopoietic transcription factors, during differentiation (Fig. 1E,F); indicating that cardiac myocyte differentiation predominates in this system. Mesp1-EYFP+ cells started to appear at day 3, continued to increase in number up to day 5 of embryoid body (EB) culture^19^. Throughout this study, we capture EYFP+ cells at day 5 as the starting point, as these cells represent a progenitor cell population with minimal signs of cardiac or other lineages commitment.

### The Mesp1-CPC lineage has a unique gene expression profile

**Figure 2:**
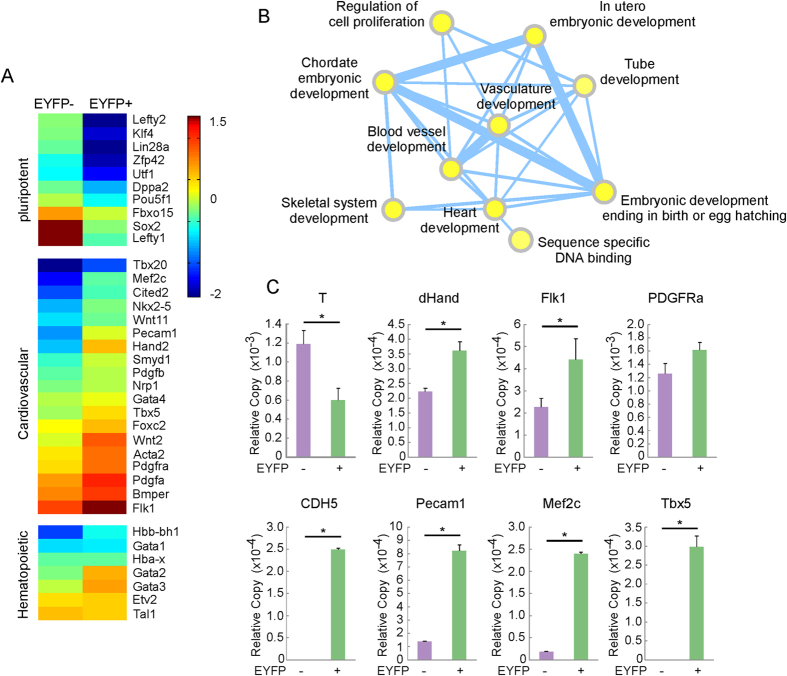
Microarray survey of global gene expression in Mesp1-CPCs. Day 5 EB culture of Mesp1Cre-EYFP cells were separated into EYFP- and + cells, and global gene expression profile was analyzed by microarray. (**A**) Selective enrichment of gene subset in EYFP+ cells. Genes associated with pluripotency; nascent mesoderm and endoderm were enriched in EYFP- cells, whereas markers of cardiovascular and hematopoietic lineages were enriched in EYFP+ cells. (**B**) Gene ontology networks enriched in EYFP+ CPCs. (**C**) Real-time RT-PCR confirmation of selected genes. N ≥ 3; *p < 0.05 versus control cells.

Even though the heart is the first organ to form in vertebrate embryos, the specification of cardiac progenitors far precedes the appearance of the cardiac crescent or paired heart tubes. To gain insight of early cardiac lineage specification, we captured day 5 Mesp1 lineage cells and studied their gene expression signature by whole genome microarray. A total of 781 unique genes were enriched in the Mesp1-EYFP+ cells compared to EYFP- cells (fold change >2, p < 0.01). GO analysis revealed the descriptors “heart development”, “vasculature development’’ and “blood vessel development” to be among the most significantly enriched GO terms (Supplemental Table 2). An independent EnrichmentMap analysis confirmed that the most significantly enriched networks in EYFP+ CPCs were centered on the aforementioned GO terms (p < 0.01, Fig. 2B). In contrast, no development-related gene ontology networks were enriched in EYFP- cells (Supplemental Figure S1). Hence, Mesp1-EYFP+ cells recapitulate the common progenitor of the cardiovascular cells.

Figure 2A shows the heat map of a few marker genes that defined the position of early Mesp1-CPCs during the course of ES cell differentiation. Pluripotent stem cell markers such as Oct4, Sox2, Klf4, Lin28a and Lefty1 were enriched in the EYFP- fraction, agreeing with the differentiated status of the Mesp1-CPCs. A few early mesoderm markers, including Wnt8a, Fgf8 and T were also enriched in the EYFP- cells, which distinguished Mesp1-CPCs from the general nascent mesoderm population. In contrast, Mesp1-CPCs expressed other established markers of the cardiac mesoderm, Flk1, PDGFa, PDGFRa, PDGFRb and Hand2. Genes that mark the irreversible stage of cardiovascular lineages, including Smarcd3 (Baf60C), Cited2, Mef2c, Smyd1, Wnt11 and Nkx2-5, were expressed at low levels, which were consistent with the differentiation stage of these cells. They were nonetheless enriched in the EYFP+ cells, suggesting early commitment of the cardiac progenitors. Quantitative RT-PCR verified enrichment of nascent mesoderm marker T and endoderm marker Sox17 in the EYFP- cells, while the Mesp1-CPCs exhibited enriched cardiac mesoderm markers dHand, Flk1 and PDGFRa (Fig. 2C). Markers of endothelial cells (CDH5 and Pecam1) and cardiac myocytes (Mef2c, Tbx5, αMHC) displayed elevated levels in EYFP+ cells, supporting that Mesp1 derived lineages mainly contribute to the cardiovascular system. EYFP+ cells also displayed markers of the hematopoietic lineages (Fig. 2A, ***bottom panel***). However, subsequent cardiac myocyte differentiation measured by cell type specific gene activity was more robust than those of hematopoietic cells (Fig. 1F). Therefore, we have captured a unique population of cardiac progenitors, which are earlier than the progenitors marked by Nkx2-5 and Isl1, but distinguished from the rest of the nascent mesoderm.

### The Mesp1 lineage is a specific subset of PDGFRA+/Flk1+ cells

**Figure 3:**
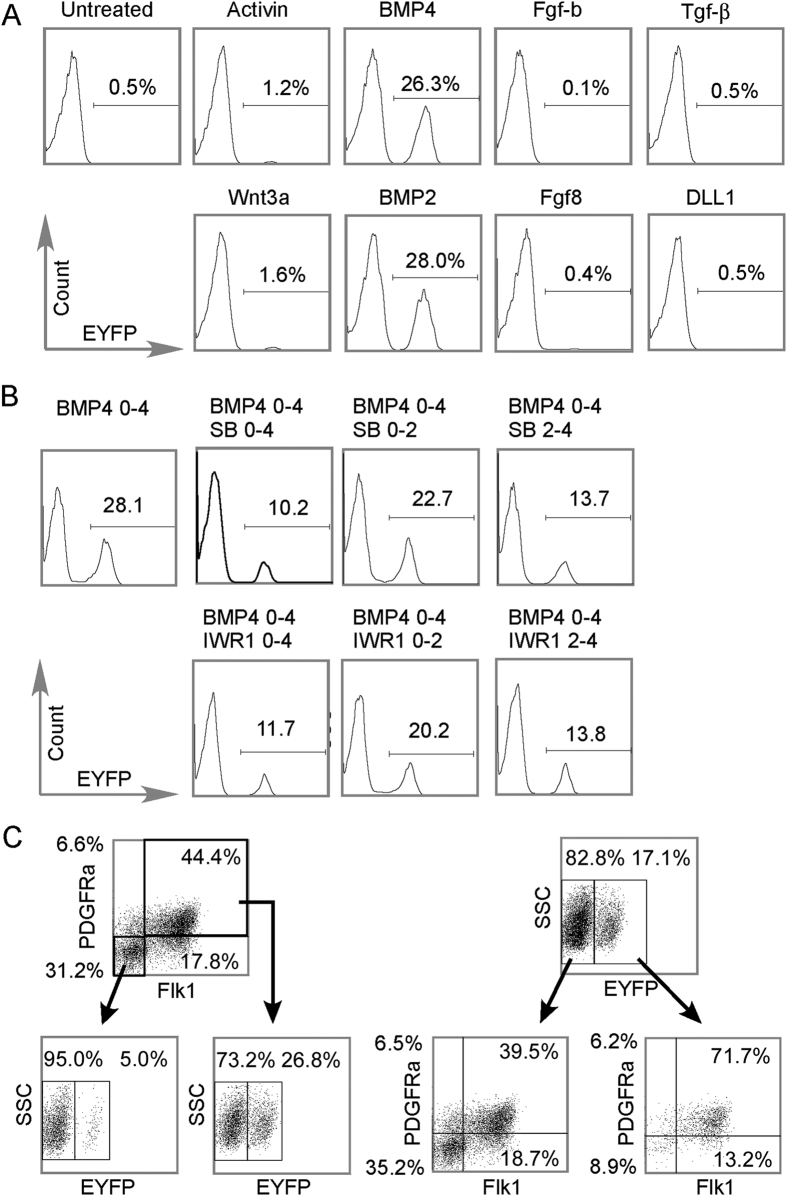
Mesp1-lineage is contingent upon BMP2/4, canonical Wnt and Nodal/Activin signals. (**A**) BMP2/4 were the most potent growth factors in driving the appearance/expansion of Mesp1-CPCs cells. Of the eight growth factors surveyed, BMP2/4 were most potent in driving the appearance/expansion of the EYFP+ population, while Activin and Wnt3a were also effective though to a much less extent. FACS was performed for day 5 EBs receiving growth factor treatments from day 0 to 4. (**B**) Nodal/Activin inhibitor SB431542(SB) and canonical Wnt inhibitor IWR1 blocked the induction/expansion of Mesp1-CPCs. Cells cultured as EBs were exposed to the treatment as indicated, then the EFYP signals were captured on day 5 by FACS. While SB431542 and IWR1 effectively blocked the induction/expansion of the EFYP+ cells when given at all-time intervals, they were more potent at the later stage (day 2–4). (**C**) Mesp1-CPCs cells represent a specialized population out of the Flk1+/PDGFR1+. Cells from day 4 EBs were dissociated, stained for both Flk1 and PDGFR1, and analyzed by tri-color FACS. Left side: cells were first analyzed for Flk1 and PDGFR1 expression, next the double negative cells (Flk1-/PDGFR1-) or double positive cells (Flk1+/PDGFR1+) were analyzed for EFYP signal. EYFP+ cells are almost exclusively from Flk1+/PDGFR1+ cells. Right side: cells were first analyzed for EYFP signal; next the EYFP- or EYFP+ cells were analyzed for Flk1 and PDGFR1 signals. EYFP- cells were close to evenly distributed into Flk1-/PDGFR1- and Flk1+/PDGFR1+ cells (35.2% vs. 39.5%). EYFP+ cells were mainly Flk1+/PDGFR1+ (71.1%).

Extracellular cues, especially the Activin/Nodal, BMP, and Wnt families, play pivot roles in establishing the pre-cardiac mesoderm. Early embryo studies across several species had established the Tgfβ family members, Activin/Nodal and BMPs, were essential for cardiogenesis^23^,^24^. While non-canonical Wnts induce cardiac specification from non-cardiac mesoderm, canonical Wnts are inhibitory^25^,^26^,^27^. In ESC differentiation studies, members of all three families were used to induce mesoderm formation^5^,^28^. However, a switch-off of these signals were proposed for the next step in cardiac differentiation^29^,^30^,^31^. Thus, we evaluated the extracellular cues that drive the Mesp1-lineage with an array of growth factors (Fig. 3A). Activin and Wnt3a marginally augmented the prevalence of the Mesp1-CPCs (1.2% and 1.6%, respectively) while BMP2 and BMP4 were the most potent (28.0% and 26.3%, respectively). Fibroblast growth factors, including Fgf-b and Fgf8, and others such as Tgf-β and the Notch ligand, Dll1, failed to increase the number of Mesp1-CPCs. But, despite a lower activity in inducing the Mesp1-CPCs, Wnt and Activin/Nodal signaling still played important roles in the determination of CPCs. BMP4 failed to fully induce the CPC population in the presence of either 5 μM Wnt inhibitor IWR1, or 10 μM Nodal/Activin inhibitor SB431542 (SB) (Fig. 3B). Furthermore, the presence of IWR1 or SB431542 at day 2-4 largely reproduced their effect at day 0-4, supporting the requirement of both Wnt and Activin/Nodal signaling at the time of cardiac progenitor cell specification, but not at a prerequisite step. These data agree with the established role of the three pathways in early cell fate determination.

We then compared the Mesp1-CPCs to a widely recognized cardiac progenitor population, the Flk1+/PDGFRa+ cells (Fig. 3B). At day 3.5 of EB culture, a time point frequently used for isolation of Flk1+/PDGFRa+ cardiac progenitors, we isolated either Flk+/PDGFRa+ or Mesp1-CPCs by FACS. In a pilot experiment, single staining was performed to ensure that each factor was detected in its assigned channel without leaking into other channels. Supplemental Figure S2 shows the detection of PE-labeled PDGFRa, APC-labeled Flk1, and Mesp1-EYFP without interference with each other. In Fig. 3B, the three factors were simultaneously detected in which Mesp1-CPCs represented 17.1% of the total population. The Mesp1-CPCs were composed of 71.1% Flk1+/PDGFRa+ marked cells, while out of non-Mesp1-lineage cells, 39.5% were also Flk1+/PDGFRa+ marked cells. Furthermore, Flk1+/PDGFRa+ cells represented 44.4% of the total population in which 26.8% were Mesp1-CPCs and very few Flk1-/PDGFRa- cells were Mesp1-CPCs. Since, Flk1+/PDGFRa+ CPCs require both BMP4 and Activin supplements^5^, we asked whether these growth factors would boost the prevalence of Mesp1-CPC. Increased amounts of BMP4 were associated with more Mesp1-CPCs, while increased dosage of Activin failed to augment the number of Mesp1-CPCs. Also, the addition of Activin with BMP4 did not further increase the yield of Mesp1-CPCs (Supplemental Figure S3). Thus, Mesp1 is a specified subpopulation out of the Flk1+/PDGFRa+ cells and BMP signaling is the most potent driver of the Mesp1-CPCs.

### Defining two distinct BMP4 dependent stages of cardiac differentiation

**Figure 4:**
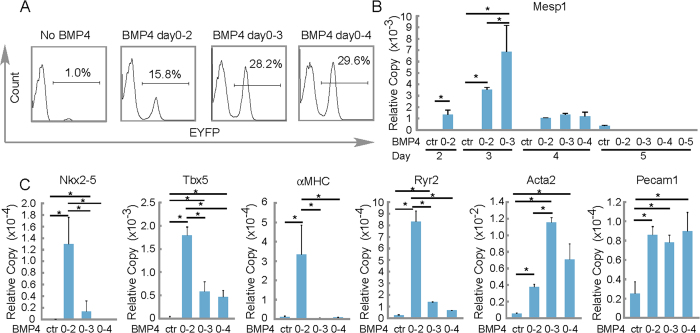
Cardiac differentiation from Mesp1-CPCs into cardiac myocytes requires precise dosage and timing exposure to the growth factor BMP4. (**A**) The number of Meps1-EYFP+ cells were positively correlated to the time exposure to BMP4. FACS was performed on day 5 EB-derived cells. (**B**) BMP4 treatment led to precocious and augmented Mesp1 expression. N ≥ 3; *p < 0.05. (**C**) Prolonged exposure to BMP4 led to reduced expression of markers of the cardiac program. EBs were collected on day 8 and gene expression was analyzed by real-time RT-PCR. N ≥ 3; *p < 0.05.

Additionally, we observed that BMP4 exposure for the first two days led to 15.8% of EYFP+ cells, day 0–3 exposure led to 28.2% of EYFP+ cells, while 0–4 exposure barely increased EYFP+ cells to 29.6%, as assayed by FACS on day 5 (Fig. 4A). By measuring Mesp1 expression with real-time RT-PCR at serial time points, we found that Mesp1 was sensitive to BMP4 induction. Exposure to BMP4 from days 0–2 was optimal for inducing precocious Mesp1 expression (Fig. 4B). Surprising, protracted exposure to BMP4 yielded more Mesp1-EYFP+ (days 0-3 or days 0-4) yet blocked the formation of cardiac myocytes and generated smooth muscle cells and endothelial cells (Fig. 4C). In contrast, BMP4 days 0–2 supplement only led to a modest increase in the percentage of the Mesp1-EYFP+ cells, but induced definitive cardiac myocyte formation. So, the prevalence of Mesp1-EYFP+ cells that may differentiate into cardiac myocytes requires attention to BMP4 dosage and timing.

**Figure 5:**
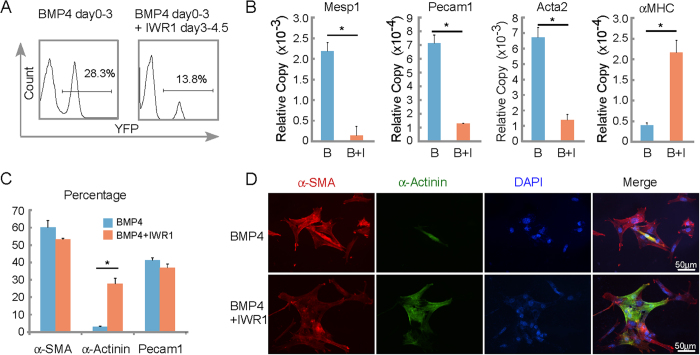
Differentiation of Mesp1-CPCs from cardiac mesoderm into cardiac myocytes requires inhibition of canonical Wnt signaling. (**A**) Canonical Wnt inhibitor IWR1 limited the appearance/expansion of Mesp1-CPCs induced by BMP4. FACS analysis was performed for day 5 EBs receiving the growth factor/ inhibitor treatment as indicated. (**B**) Canonical Wnt inhibitor IWR1 (I) induced the cardiac program at the cost of other cardiovascular lineages. Mesp1, in the presence of BMP4 (**B**), Pecam1, Acta2 (alpha-smooth muscle actin), and αMHC were assayed by real-time RT-PCR. *p < 0.05 versus control cells; N ≥ 3. (**C**) Canonical Wnt inhibitor IWR1 induced the cardiac myocyte formation. Cells were stained by antibodies indicated. The percentages were calculated as positive staining vs. nuclei. (**D**) Canonical Wnt inhibitor IWR1 induces the formation of cardiac myocytes from α-SMA positive cells.

Canonical Wnt inhibitors are known to convert cardiac mesoderm to cardiac myocytes^32^,^33^,^34^,^35^,^36^ and their role was investigated during the differentiation of Mesp1-CPCs. As described, BMP4 days 0–3 supplement led to higher percentage (∼28%) of Mesp1-CPCs, albeit with fewer cardiac myocytes. In contrast, the BMP4 treatment followed by Wnt inhibitor IWR1, reduced the percentage (13.8%) of Mesp1-CPCs, but allowed them to differentiate more efficiently into cardiac myocytes (Fig. 5A). In addition, Mesp1 mRNA was reduced along with markers of vascular endothelium (Pecam1) and smooth muscle cells (Acta2) while cardiac αMHC expression was augmented (Fig. 5B); Sequential exposure to BMP4 and IWR1, EYFP+ CPCs did not significantly reduce the percentage of SMCs or ECs, but expanded the appearance of cardiomyocytes (Fig. 5C). In fact, BMP4 plus IWR1 treatment yielded cardiac myocytes and beating clusters than BMP4 days 0–2 exposure (not shown), despite a similar number of EYFP+ cells, which suggests that inhibition of canonical Wnt signaling has additional roles in facilitating the appearance of cardiomyocytes than just limiting CPC expansion. As shown in Fig. 5D α-Actinin positive cells arose from α-smooth muscle actin stained cells, and did so much more efficiently in the presence of IWR1. The Mesp1-CPC exhibits dynamism in the transition between cardiac mesoderm to cardiac myocyte that likely require very different set of gene regulation networks: the first step comprises the induction of the Mesp1-CPC; and the second step comprises the conversion of such cells to cardiac myocytes.

### Mesp1-CPCs differentiated into the three major cardiovascular cell types in the myocardium of post infarcted hearts

**Figure 6:**
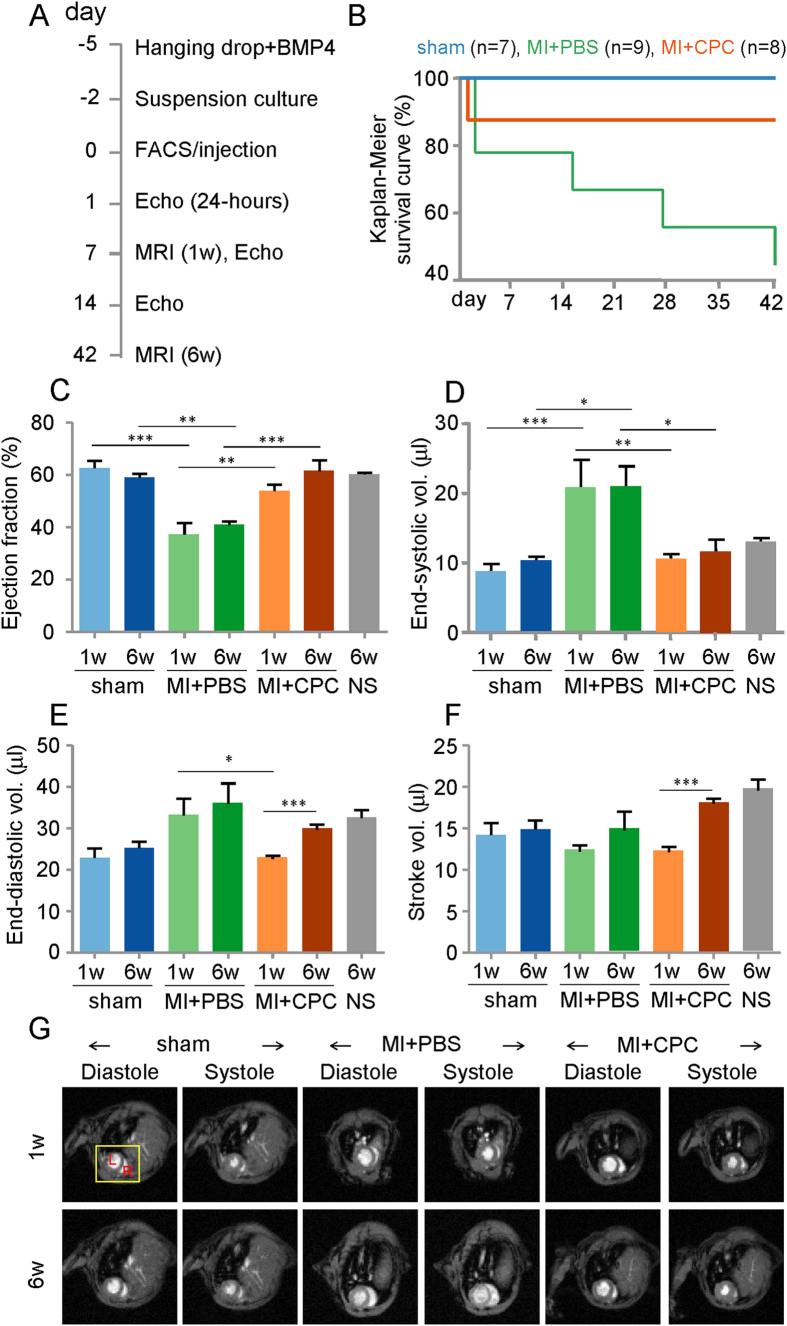
Injection of Mesp1-CPCs into post-MI hearts led to improved cardiac function and survival. (**A**) The timeline of the study. (**B**) Improved survivability of MI hearts injected with Mesp1-CPCs. Kaplan-Meier survival analysis of mouse cohorts. Mice were monitored daily for survival data analysis. The cohorts included sham-operated control mice (n = 7), myocardial infarcted mouse injected with PBS media (MI + PBS) control mice (n = 9), and myocardial infarcted mouse injected with Mesp1-CPCs (MI + CPCs) experimental mice (n = 8); p < 0.05, Log-rank (Mantel-Cox) test. (**C–G**) Restored cardiac function of MI hearts injected with Mesp1-CPCs. Cardiac magnetic resonance imaging (MRI) of mice at 1 week and 6 weeks following induction of MI plus intramyocardial injection of the CPCs. Control groups include sham-operated mice, MI-operated mice injected with PBS and un-operated control mice. (**C**) Left ventricular ejection fraction (LV-EF). (**D**) Left ventricular end-systolic volume (LV-ESV). (**E**) Left ventricular end-diastolic volume (LV-EDV). (**F**) Left ventricular stroke volume. (**G**) MRI images during systole and diastole at 1 week and 6 weeks post-MI. Data are expressed as the mean ± S.E.M.; sham (n = 6; 1-week) and (n = 7; 6-weeks), MI+PBS (n = 6; 1-week) and (n = 4; 6-weeks), MI+CPC (n = 7; 1-week) and (n = 7; 7-weeks); NS = No Surgery controls (n = 4; 6-weeks); *p < 0.05, **p < 0.005, ***p < 0.0005.

In a blinded study, day 5 Mesp1-CPCs were injected into the intramyocardial tissue within the infarct and border zone areas, immediately following myocardial infarction (MI) induction by left anterior descending artery (LAD) ligation (Fig. 6A). We verified the degree of myocardial infarction was both comparable between MI+PBS control and MI+CPCs experimental groups as well as had expected similarly depressed cardiac function, as measured by the echocardiographic parameters of percent left ventricular fractional shortening (%LV-FS) and percent left ventricular ejection fraction (%LV-EF), at 24-hours post-MI induction (Supplemental Figure S4; Supplemental Table 3). In addition, we verified no differences in cardiac function assessment between sham-operated and un-operated control animals (Fig. 6C–F). The Kaplan-Meier survival curve showed that the injected Mesp1-CPCs into the infarcted myocardium improved the survivability of the animals over 42 days (6 weeks) of the study as compared to the infarcted myocardium without the injected Mesp1-CPCs (Fig. 6B). Next, we assessed cardiac function by using MRI at 1 week and 6 weeks following induction of the MI. Usually, following an MI the heart is unable to eject sufficient volume of blood to meet the animal’s needs. The failing heart compensates by adjusting the LV-ESV, the amount of blood remaining in the LV at the end of systole (contraction) and by adjusting the LV-EDV, the amount of blood in the LV at the end of diastole (relaxation). We observed an increase in the LV-ESV in the MI+PBS control mice versus sham-operated mice, at both 1 week and 6 weeks. The increased LV-ESV was reversed in infarcted mice injected with Mesp1-CPCs, at both time points (Fig. 6D). Also, there was a trend for increased LV-EDV in the MI+PBS control mice versus sham-operated mice at both time points, however we did observe a significant decrease in LV-EDV in infarcted mice injected with the Mesp1-CPCs versus infarcted mice without CPCs (Fig. 6E). The percent left ventricular ejection fraction (%LV-EF), directly measured by MRI, is another important measure of cardiac function that represents the total amount of blood within the LV that is then pumped out of the heart during each contraction. We observed similar %LV-EFs in sham-operated control mice at 1 week (63%) and 6-weeks (59%) post the sham surgery versus un-operated control mice (60%), thus showing the reliability of the cardiac MRI to assess function (Fig. 6C). Also as expected, the %LV-EF of the MI+PBS control mice was significantly reduced versus sham-operated mice at 1-week post-MI (37%). Reduced cardiac function persisted for up to 6 weeks post-MI of the study (41%), thus demonstrating the validity of our ischemic MI model. In contrast, infarcted hearts injected with the Mesp1-CPCs, showed improved cardiac %LV-EF at 1 week post-MI (54%) that was significantly greater than infarcted hearts without the CPCs. Improved %LV-EF remained up to 6 weeks post-MI (62%), the end of the study (Fig. 6C), and was not significantly different than the %LV-EF of sham-operated mice (Fig. 6C). The stroke volume, which represents the volume of blood pumped from the LV with each heart beat and calculated as the LV-EDV minus the LV-ESV, significantly improved at 6 weeks compared to 1 week in post-infarcted mice injected with the Mesp1-CPCs (Fig. 6F). Restoration of cardiac function of infarcted hearts injected with Mesp1-CPCs was also visualized by cardiac MRI videos (Fig. 6G; Supplemental Videos 1, 2, 3, 4, 5, 6).

We next determined the effect of the Mesp1-CPCs on scar formation and infarct size. Consistently, decreased but variable scar formation was also observed in the serial Hematoxylin and Eosin (H&E) sections of infarcted hearts injected with Mesp1-CPC (Supplemental Figure S5; *boxed areas*) as compared to PBS injected hearts. Furthermore, decreased infarct size was also observed from serial Masson Trichrome (MT) sections of infarcted hearts injected with Mesp1-CPC (Supplemental Figure S6). At 6 weeks post-MI, the scar size was 30.1 ± 9.5% in the MI+PBS control versus 19.5 ± 5.3 (p < 0.05) in the MI+CPCs experiment groups. In a few animals (n = 3) that were kept alive until 12-weeks post-MI, the scar size was 25.5 ± 4.7% in the MI+PBS control versus 8.6 ± 3.4 (p < 0.05) in the MI+CPCs experiment groups. No scar formation was observed in the sham-operated group at either 6 weeks or 12 weeks post-MI induction. Thus, it’s likely that transplanted mouse pre-contractile Mesp1-EYFP+ CPCs showed homing to the site of cardiac injury, improved the survivability of these injured mice and restored the functional performance of the myocardial infarcted hearts.

**Figure 7:**
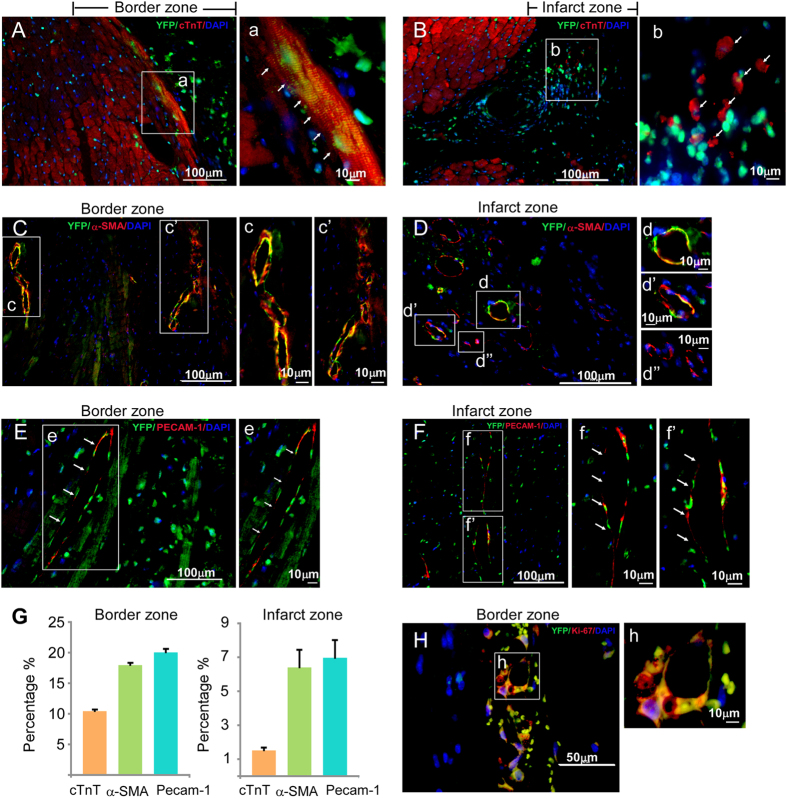
Survival and differentiation of Mesp1-CPCs in post-MI hearts. (**A**) EYFP+ CPCs contribute to cardiac myocytes in the border zone. (**B**) EYFP+ CPCs cannot differentiate into sarcomeric cardiac myocytes in the infarct zone. (**C**) EYFP+ CPCs differentiate into vascular smooth muscle cells in the border zone. (**D**) EYFP+ CPCs differentiate into vascular smooth muscle cells in the infarct zone. (**E**) EYFP+ CPCs differentiate into vascular endothelial cells in the border zone. (**F**) EYFP+ CPCs differentiate into vascular endothelial cells in the infarct zone. (**G**) The distribution of CM, SMC and EC differentiation from EYFP+ cells in post-MI hearts. (**H**) EYFP+ CPCs are proliferative in the border zone. In control experiments, we did not observed any GFP+/Ki67+ cells within the border zone of post-MI hearts that were injected with MI+PBS (Supplemental Figure S8).

Did injected Mesp1-EYFP+ CPCs differentiate into CMs, SMCs and ECs in the post-MI hearts? After 3 months, infarcted regions of the hearts were sectioned and stained for EYFP to detect the injected Mesp1-CPCs and their terminally differentiated progeny: cardiac myocytes, smooth muscle and endothelial cells with anti-cTnT, α-SMA and Pecam1. The majority of EYFP/cTnT double stained cells were found in the border zone (Fig. 7A). Characteristic well-organized myofibrillar striations confirmed identity of *de novo* cardiac myocytes (Fig. 7A,E). EYFP/cTnT double positive cells were rarely found in the infarct zone, a microenvironment incompatible for generating myocytes (Fig. 7B). In contrast, EYFP/α-SMA double positive cells were found in the border zone, as well as the infarct zone and frequently associated with “ring” structures indicating CPC-derived smooth muscle cells contributed to neovasculogenesis (Fig. 7C,D). Similarly, EYFP/Pecam1 double positive cells, as well as EYFP/α-SMA positive cells were found in both border and infarct zones, and associated with “ring” structures, which indicated the incorporation of CPC-derived endothelial cells into *de novo* vasculature in post-MI hearts (Fig. 7E,F) and increased vasculogenesis. Identification of Mesp1-EYFP+ cells in the post-MI hearts was also further demonstrated by staining the infarct/border zone with the anti-EYFP antibody followed by non-fluorescence visualization (Supplemental Figure S7). A summary of the distribution of terminal differentiated cells derived from injected CPCs, indicated more SMCs and ECs than CMs were formed in post-MI hearts (Fig. 7G). Furthermore, the majority of EYFP+ CMs of MI+CPC hearts were located at the border zone of the infarct. The percentage of EYFP+ CMs/LV in total was 0.50 ± 0.01% (n = 3), in the MI+CPCs heart samples. However, we also observed that the percentage of EYFP+ SMCs/LV in total was 9.74 ± 0.20% (n = 3) and EYFP+ ECs/LV in total was 10.80 ± 0.31% (n = 3), in the MI+CPC heart samples. Therefore, we believe that although the regeneration of cardiomyocytes contributed to the recovery of cardiac function in the MI+CPC hearts, the major recovery factors more likely includes the reperfusion of blood flow to the border zone within the infarcted area as well as the paracrine effects that was induced by the coronary vasculature regeneration from the EYFP+ CPCs. In addition, we often observe the co-localization of EYFP and Ki67 with the “ring” structure of neovasculature, suggesting that CPC-derived cells were in active cycling (Fig. 7H). In control experiments, we did not observed any GFP+/Ki67+ cells within the border zone of post-MI hearts that were injected with MI+PBS (Supplemental Figure S8). Thus, transplanted mouse pre-contractile Mesp1-EYFP+ CPCs directly differentiated into CMs, SMCs and ECs, and contributed to the neovasculature in the post-MI hearts.

## Discussion

Here, we generated ES cell lines with a Mesp1-triggered permanent EYFP signal to trace cardiac progenitor cells. Earlier reports allowed the isolation of Mesp1-CPCs, but further tracking of these cells was not possible, because of the transient nature of Mesp1 expression^37^,^38^. Our approach is an advance for the following reasons: 1) The knock-in Cre in the Mesp1 locus ensured better fidelity over transgene based approaches; 2) The Cre-LoxP strategy converted the transient Mesp1 expression into a permanent EYFP lineage tracer, making it possible to trace the expansion and differentiation of CPCs *in vitro* and *in vivo*; 3) Compared to the low prevalence of Mesp1-GFP cells (∼2%)^39^, we were able to obtain >10 fold more (∼28%) EYFP+ cells with BMP2/4 supplementation. This difference might reflect the rapid expansion of this population in the early developmental stage. As the cells retain the signature of early CPCs, this strategy allowed us to obtain enough material for the study of cell transplantation in a post-MI mouse model.

BMP2/4 were the most potent growth factors to drive the expansion of the Mesp1-CPCs, in the absence of Activin. This is in line with the previous finding that BMP2 drives the SSEA1+ CPC population^40^,^41^. BMP4 in combination with Activin was shown to drive the prevalence of Flk1+/PDGFRa+ population but such a combination failed to generate more Mesp1-CPCs, than BMP4 alone. Perhaps, Activin may be more important in driving non-Mesp1 CPCs, or driving other mesoderm lineages. Crosstalk between the signaling pathways from BMP2/4 to Wnt, Nodal/Activin is required for the generation of CPCs. As documented in this study, the presence of either Wnt inhibitors or Nodal/Activin inhibitors partially abolished the effect of BMP4.

Flk1+/PDGFRa+ cells are perhaps the most widely used CPCs, but Mesp1-CPCs represent a further enriched progenitor population for the cardiovascular system. The gene expression profiles of Mesp1-CPCs support this notion, as shown in Fig. 2. Genes specific for stem cells and nascent mesoderm, such as T, were selectively enriched in the EYFP- population. Markers of the cardiovascular lineages, which at the time of capture, had just started to appear, were highly enriched in the EYFP+ population; thus we placed Mesp1-CPCs in the developmental hierarchy between the Flk1+/PDGFRa+ population and the Nkx2-5+ and Isl1+ populations, all of which are downstream of SSEA1+ CPCs^40^,^41^.

Our study showed the interplay between BMP4, prevalence of Mesp1-CPCs and their developmental outcome. Prolonged BMP4 exposure led to more Mesp1-CPCs but their conversion to cardiac myocytes was negatively impacted. For example, shorter exposure to BMP4 led to less Mesp1-CPCs, but more cardiac myocytes, while lengthy BMP4 exposure appeared less influential on smooth muscle and endothelial cell differentiation. The transition from Mesp1-CPCs towards cardiac myocytes likely requires a sharp change in BMP4 signaling. Biphasic requirements of extracellular cues have set a precedent in cardiopoesis. Canonical Wnt signaling is essential for the process of cardiac mesoderm formation but needs to be switched off prior to the formation of cardiac myocytes^32^,^33^,^34^,^35^,^36^. In this study, the presence of Wnt inhibitor IWR1 led to significantly more cardiomyocytes, consistent with the biphasic role of canonical Wnts. Recently, the Nodal/Tgfb signaling pathway was also found to exert a biphasic role in cardiogenesis^42^. The expansion of CPCs by core gastrulation signals and the differentiation of CPCs by the related inhibitors seem to mark two distinctive stages of cardiogenesis. Clearer definition of these biphasic control mechanisms should assist in developing *in vitro* strategies for directed differentiation towards the cardiac myocyte lineage. It’s worth noting that our conclusions are based on the theory that the major cardiovascular cell types are of an Mesp1+ origin. However, a Mesp1-dependent step may be by-passed^31^,^43^. Whether such alternative differentiation routes require the same dynamic control of extracellular cues cannot be addressed here, as the YFP- fraction may contain significant amount of Mesp1-lineage cells that are not YFP+ due to Cre efficiency variability.

In a post-MI mouse model, the Mesp1-CPCs differentiated into cardiac myocytes, vascular smooth muscle and endothelial cells. CPC-injected post-MI mice showed marked improvement of cardiac pump function and overall rate of survival. Cardiac myocyte differentiation was restricted to the infarct/border zone, and of lower prevalence than the other two lineages. CPC-derived vascular smooth muscle and endothelial cells were widely present in both infarct zone and infarct/border zones. They frequently contributed to neovasculogenesis, and remarkably, co-stain with markers of proliferation. Thus, we feel that the functional improvement and better survival of CPC-injected mice may be tilted more by neovasculargenesis than cardiac myocyte restoration. Further functional improvement may be possible with increased cardiac myocyte differentiation in the infarct zone, which in turn may benefit from conditioning the toxic environment of the infarct zone by surviving factors such as SDF-1, or providing pro-cardiac myocyte factors such as locally administrated canonical Wnt inhibitors.

Recently, Chan *et al*. described that Mesp1 lineage cells contributed to hematopoietic and skeletal muscle in addition to cardiovascular lineages^44^. We also observed EYFP+ cells in extraembryonic tissues in the early embryo (Data not shown). Chan *et al*. used forced Mesp1 expression to show context-dependent differentiation towards non-cardiac cells, indicating the high plasticity of the early progenitor cells. However, since Mesp1 expression normally appears much earlier than the appearance of their gene target, Tal1 in differentiated EBs; making it less likely that Mesp1 directly targets the blood program. Also, Mesp1-CPCs contribute primarily to cardiovascular lineages and showed much greater cardiac transcription factor expression compared to hematopoietic transcription factors; so we feel that Mesp1 is a specific driver of CPCs.

In short, we have developed a valuable ES cell model to trace the fate of CPCs. They possess a unique gene expression signature, which may be important for regulating fate-determining factors at the early stage of cardiogenesis. The response to BMP4 distinguished two stages, CPC expansion and differentiation, in cardiogenesis. CPCs hold unique promise in cell therapy for heart diseases: they are fate-restricted cells and are not hyperproliferative as embryonic stem cells, yet they are still multipotent and contribute to all the major cell lineages of the cardiovascular system.

## Summary

Mesp1 is the earliest driver of the cardiovascular lineages. How the progeny of Mesp1-expressing cells contribute to multiple lineages in ES cell differentiation and heart diseases has not been explored, due to the transitory nature of its expression. Here, we describe the isolation and characterization of Mesp1-CPCs. They are a more specific subpopulation of the widely used Flk1+/PDGFRa+ cells, and express previously uncharacterized mRNA and miRNA signature. Expansion and cardiac differentiation of these cells take abrupt change of extracellular cues, thus defining clear steps for directed programming. Most importantly, these cells differentiated into vascular smooth muscle cells, endothelial cells and cardiac myocytes in post-MI mouse hearts, and remained proliferative at 12-week post injection. The Mesp1- CPCs system described here offers a new platform and resource to explore the specification and differentiation of cardiovascular cells, and their use in repairing injured hearts.

## Additional Information

**How to cite this article**: Liu, Y. *et al*. Mesp1 Marked Cardiac Progenitor Cells Repair Infarcted Mouse Hearts. *Sci. Rep*. **6**, 31457; doi: 10.1038/srep31457 (2016).

## Supplementary Material

Supplementary Information

Supplementary Movie S1

Supplementary Movie S2

Supplementary Movie S3

Supplementary Movie S4

Supplementary Movie S5

Supplementary Movie S6
