# Supplementary material for: Mesp1 Marked Cardiac Progenitor Cells Repair Infarcted Mouse Hearts
Source: Sci Rep. 2016 Aug 19;6:31457. doi: 10.1038/srep31457 (PMC4990963; doi:10.1038/srep31457)
Supplement: Supplementary Information [file srep31457-s1.pdf]

Supplemental Information

Running Title: CPCs Repair Post-MI Hearts

Mesp1 Marked Cardiac Progenitor Cells Repair Infarcted Mouse Hearts

Yu Liu [1,*], Li Chen [1,*], Andrea Diaz Diaz [2], Ashley Benham [1], Xueping Xu [3], Cori S. Wijaya [2], Faisal Fa'ak [2], Weijia Luo [1], Benjamin Soibam [1], Alon Azares [4], Wei Yu [1], Qiongying Lyu [1,#], M. David Stewart [1], Preethi Gunaratne [1], Austin Cooney [3], Bradley K. McConnell [2,*,†], Robert J. Schwartz [1,4,†]

* These authors contributed equally to this work.

[1] Department of Biology and Biochemistry, University of Houston, Houston, TX 77204

[2] Department of Pharmacological and Pharmaceutical Sciences, University of Houston, Houston, TX 77204

[3] Department of Molecular and Cellular Biology, Baylor College of Medicine, Houston, TX 77030

[4] Stem Cell Engineering, Texas Heart Institute at St. Luke's Episcopal Hospital, Houston, TX 77030

[#] Current address: Department of Obstetrics and Gynecology, Renmin Hospital of Wuhan University, Wuhan, Hubei, 430060, China

List of Supplemental Files.

Supplemental Methods.

Supplemental Figure Legends.

Supplemental Table 1. RT-PCR primers and probes list.

Supplemental Table 2. GO terms selectively enriched in Mesp1-CPCs.

Supplemental Table 3. Echocardiographic characteristics of injected Mesp1-CPCs post-MI hearts.

Supplemental Figure S1. Gene ontology networks negatively enriched in Mesp1-CPCs.

Supplemental Figure S2. Establishment of the method to accurately detect Flk1, PDGFRa and Mesp1-EYFP+ cells by FACS.

Supplemental Figure S3. Activin A did not cooperate with BMP4 in inducing/expanding Mesp1-CPCs.

Supplemental Figure S4. Percent fractional shortening (LVFS%) and percent ejection fraction (LVEF%) of injected Mesp1-CPCs post-MI hearts.

Supplemental Figure S5. H&E serial sections of injected Mesp1-CPCs post-MI hearts show improved infarct repair.

Supplemental Figure S6. Masson trichrome staining of injected Mesp1-CPCs post-MI hearts show improved infarct repair at 12 weeks.

Supplemental Figure S7. Identification of Mesp1-CPCs (EYFP+) cells in post-MI heart.

Supplemental Figure S8. Border zone of MI+PBS in post-MI hearts show no GFP+/Ki67+ cells (*negative control for Figure-7H*).

Supplemental Video 1. Cardiac MRI of a mouse at 1 week following sham-operation (ID#6LL).

Supplemental Video 2. Cardiac MRI of a mouse at 6 weeks following sham-operation (ID#6LL).

Supplemental Video 3. Cardiac MRI of a mouse at 1 week following induction of MI-operated mice injected with PBS (ID#12LL).

Supplemental Video 4. Cardiac MRI of a mouse at 6 weeks following induction of MI-operated mice injected with PBS (ID#12LL).

Supplemental Video 5. Cardiac MRI of a mouse at 1 week following induction of MI plus intramyocardial injection of Mesp1-CPCs (ID#1LR).

Supplemental Video 6. Cardiac MRI of a mouse at 6 weeks following induction of MI plus intramyocardial injection of Mesp1-CPCs (ID#1LR).

Supplemental Methods.

Myocardial Infarction (MI) Surgery.

An animal model of ischemic myocardial infarction (MI) in 8-10 week old adult male C.B-17 SCID mice was induced by chronic (permanent) ligation of the left anterior descending (LAD) artery (McConnell et al., 2009) and as we have similarly performed in rats (Wu et al., 2011). Prior to surgery, all instruments were sterilized in a dry bead sterilizer and the animal was prepared for sterile surgery. Anesthetized mice (2% isoflurane in 100% oxygen) were placed in a supine position and an endotracheal polyethylene tube (BD Intramedic™ Polyethylene Sterile Tubing, PE 90) was inserted into the trachea, visually guided by use of a dissecting microscope. Once proper position was confirmed, the endotracheal cannula was then connected to a volume-cycled rodent ventilator (Harvard Apparatus Inspira Advanced Safety Ventilator), which was supplemented with 100% oxygen and with a tidal volume approximately 0.15 - 0.25 ml and a respiratory rate of 100 - 125 breaths per minute. Once steady breathing was established, and deep anesthesia was confirmed by the limb withdrawal response and then placed onto a heated surgical table and draped to prevent contamination from the area. Thoracotomy was performed by a small incision at the third intercostal space and the intercostal muscles were separated and dissected to the side in order to expose the ribs. Self-retaining micro-retractors were placed to separate the third and fourth ribs in order to expose the heart and visualize the LAD artery. An 8.0-prolene suture (Ethicon, Johnson & Johnson) was used to ligate the LAD artery (double ligation) at 1.5 mm distal to the left atrial appendage, which was identified directly following bifurcation of the left main coronary artery. Occlusion of the LAD artery was confirmed by observing blanching (pale coloring) of myocardial tissue distal to the suture and dysfunction of the anterior wall motion. Sham- operated control animals underwent the identical procedure, but without the ligation. Un- operated control animals did not undergo surgery. Immediately after LAD ligation, CPCs were injected within the myocardium. Control animals, that underwent LAD ligation, were injected with Phosphate Buffered Saline (PBS) within the myocardium. The rib cage was then closed with 5.0 prolene RB-1 suture (Suture Express) and the retracted muscles were placed in position and the skin was closed using 5.0 sutures. Animals were extubated and allowed to recover from anesthesia in a warm space under observation. The animals were closely monitored for any abnormal signs of pain or labored breathing before being returned to clean housing and the animal room.

Intramyocardial Injection of Mesp1-CPCs.

A mouse model of ischemic MI in 8-10 week old adult male C.B-17 SCID mice was induced by permanent ligation of the LAD artery, as described above. Ligation was verified by immediate myocardial blanching and anterior wall dysfunction. All animals with anterior wall motion dysfunction consistent with the infarct were randomized into the various groups: (1) sham-operated, (2) MI + PBS, (3) MI + Mesp1-CPCs, and (4) un-operated-controls. CPCs were injected using 5 (2 μl) injections (total volume = 10 μl) each with 20,000 CPCs (total number of cells = 0.1x106 cells) into the infarct and border zone areas, immediately following LAD artery ligation. Control groups included sham-operated mice, MI-operated mice injected with PBS and un-operated control mice. Following CPC injection, the chest was closed and the animals were monitored closely. An echocardiogram was performed 24-hr post MI to determine the extent of the induced MI. In selected mice, CPCs in mouse hearts were imaged live using bioluminescence at 24-hr post-cell injection.

Cardiac Magnetic Resonance Imaging (MRI).

Cardiac function was determined by magnetic resonance imaging (MRI) using the non-invasive Bruker PharmaScan® 7T small animal Scanner at 1-week and 6-weeks following induction of MI and / or MI plus the intramyocardial injection of the CPCs. Mice were initially anesthetized with 4–5% isoflurane (mixed with oxygen) and maintained with 1–2% isoflurane during imaging. An animal-monitoring system (SA Instruments, Stony Brook, NY) was used to monitor the mouse's ECG, respiratory rate, and body temperature. The temperature of the MRI's bore was maintained at 35°C in order to ensure sufficient and constant heart rate. Respiratory- and cardiac-gated images were acquired at end-diastole and end-systole (Costandi et al., 2007). The imaging parameters to acquire cardiac- and respiratory-gated spin echo images were as follows: repetition time (TR), 5.7 ms; echo time (TE), 2.80 ms; field of view, 4.0 cm; number of slices, 10; slice thickness, 1.0 mm; matrix, 128 × 128; and number of averages, 1. The multi-slice scan was performed in the axial orientation to visualize the left and right ventricles, and data were analyzed by using Amira 3D image processing software (Mercury Computer Systems, Chelmsford, MA). The areas representing the left and right ventricular space in each slice were summated in the amount of pixels. The volume size of the space was calculated by using the known volume size of each pixel (30.0 mm / 256 × 30.0 mm / 256 × 1.0 mm). Left ventricular (LV) epicardial and

endocardial borders were manually traced in order to calculate the LV end-diastolic volume (LV-EDV) and LV end-systolic volume (LV-ESV) using NIH ImageJ software. Total LV end-diastolic and end-systolic volumes were calculated as the sum of all slice volumes. Stroke volume (SV) was calculated according to the equation: SV = LV-EDV – LV-ESV. Ejection fraction (EF) was calculated according to the equation: LV-EF (%) = [(LV-EDV – LV-ESV) / LV-EDV] x 100. Data acquisition was performed by an experienced technologist and data analysis was measured by individuals that were blinded to the study's assignment groups.

Transthoracic Echocardiography.

Cardiac function and dimensions were evaluated by two- dimensional transthoracic echocardiography using a VisualSonics (Ontario, Canada) Vevo770 high-resolution imaging system equipped with an RMV-707B transducer (30 MHz). Animals were anesthetized by a nose cone with 1-2% isoflurane supplemented with 100% oxygen, dispensed from a calibrated precision vaporizer and with the exhaust being directed to a carbon filter scavenger. M-mode tracings of the left ventricle (LV) were used to measure left ventricular anterior wall (LV-AW) thickness, left ventricular internal diameter (LV-ID), and left ventricular posterior wall (LV-PW) thickness, both at end diastole (d) and at end systole (s). Fractional shortening (FS) was calculated according to the equation: LV-FS (%) = [(LV-ID(d) – LV-ID(s)) / LV-ID(d)] x 100. Ejection fraction (EF) was calculated according to the equation: LV-EF (%) = [(LV-Vol(d) – LV-Vol(s)) / LV-Vol(d)] x 100; where the LV chamber volume (LV-Vol) was derived from the Teichholz formula: LV-Vol(d), left ventricular volume diastole (ul) = ((7.0 / (2.4 + LV-ID(d)) * LV-ID(d)^3 and LV-Vol(s), left ventricular volume systole (ul) = ((7.0 / (2.4 + LV-ID(s))) * LV-ID(s)^3. Ligation of the LAD artery, used to induce the MI, was confirmed by echocardiography. A decreased %LV-FS of less than 25%, at 24-hours post-MI versus baseline, was excluded from the study. Data acquisition was performed by an experienced sonographer and data analysis was measured by individuals that were blinded to the study's assignment groups.

Histology and Immunohistochemistry.

Hearts were collected from euthanized animals for histological and immunohistochemical studies. Animals were first heparinized, to prevent blood clotting prior to being anesthetized

with 3% isoflurane, for 10 mins. The chest cavity was opened and the heart was excised, connective tissue removed, rinsed in PBS, blotted and weighted. The heart weights (HW) and body weights (BW) were measured and the HW to BW ratio was calculated, as a measure of cardiac hypertrophy.

For infarct size measurements, the hearts were harvested, sectioned, and stained with Masson trichrome to visualize the infarct scar. All histological sections were examined with a Nikon Eclipse microscope using a 4x objective lens. Images were captured and measured the lengths and areas of infarct and LV of each treatment group (n=3/group). The scar was measured in each section by an investigator who was blinded to the identity of the sections using the area measurement approach. Specifically, infarct scar area and the total area of LV myocardium were traced manually in the digital images and measured by the computer. Infarct size, expressed as a percentage, was calculated by dividing the sum of infarct areas from all sections by the sum of LV areas from all sections (including those without infarct scar) and multiplying by 100.

For single immunofluorescence staining, we fixed embryos in 4% paraformaldehyde/PBS, dehydrated and embedded them in paraffin for histological sections. For double immunofluorescence staining, we retrograde perfused the harvested adult mouse hearts through the aorta using a Langendorff perfusion apparatus with 10% formalin for 15 mins. Then heart samples were dehydrated and embedded in paraffin for histological sections. For antigen retrieval, we boiled sections in sodium citrate buffer (pH 6.0) for 10 mins. Sections were blocked in 2% goat serum/PBS and incubated with primary antibodies anti-GFP (Santa Cruz) and anti-cardiac troponin T (Lab Vision), anti-alpha-Smooth Muscle Actin (Sigma), anti-PECAM-1 (Abcam) or anti-Ki67 (Abcam) in blocking solution overnight at 4ºC. Then sections were treated with Alexa fluor® 488 anti-rabbit secondary antibody (Invitrogen) and Alexa fluor® 594 anti-mouse secondary antibodies (Invitrogen) at RT for 1 hr. Sections were mounted with Vectashied® with DAPI (Vector Laboratory) and photographed under a Nikon Eclipse Ti invert microscope.

For immunohistochemistry staining, we boiled sections in sodium citrate buffer (pH 6.0) for 10 mins for antigen retrieval. After peroxidase blocking (3% H_2O_2 in PBS), sections were blocked in 2% goat serum/PBS and incubated with primary antibodies anti-GFP (Santa Cruz) overnight at 4ºC. Then sections were treated with biotinylated anti-rabbit secondary

antibodies (Vector Laboratories) at RT for 1 hr, followed by treatment with Vectastain Elite ABC reagent (avidin horseradish peroxidase; Vector Laboratories). Horseradish Peroxidase (HRP) activity was revealed using the DAB kit (Vector laboratories). Sections were dehydrated, counter-stained with hematoxylin as needed, mounted with Permount and examined under a Nikon Eclipse Ti invert microscope.

Ethics Statement - Study Approvals and Consent.

All animal studies have been approved by the Institutional Animal Care and Use Committee (IACUC) and ethics committee at the University of Houston (UH; #UH-ACP-13-022) and the Baylor College of Medicine (BCM; #BCM-AN-5199). Animal care was provided in Association for Assessment and Accreditation of Laboratory Animal Care (AAALAC) accredited animal barrier facilities at UH and BCM located within the Texas Medical Center (TMC) and have therefore been performed in accordance with the ethical standards laid down in the 1964 Declaration of Helsinki and its later amendments. Also, all authors of this report gave their informed consent prior to their inclusion in the study.

Supplemental References.

Costandi, P.N., McCulloch, A.D., Omens, J.H., and Frank, L.R. (2007). High-resolution longitudinal MRI of the transition to heart failure. Magn Reson Med *57*, 714-720.

McConnell, B.K., Popovic, Z., Mal, N., Lee, K., Bautista, J., Forudi, F., Schwartzman, R., Jin, J.P., Penn, M., and Bond, M. (2009). Disruption of protein kinase A interaction with A-kinase- anchoring proteins in the heart in vivo: effects on cardiac contractility, protein kinase A phosphorylation, and troponin I proteolysis. J Biol Chem *284*, 1583-1592.

Wu, Y., Yin, X., Wijaya, C., Huang, M.H., and McConnell, B.K. (2011). Acute myocardial infarction in rats. J Vis Exp.

Supplemental Tables and Figure Legends.

Supplemental Table-S1. Taqman realtime RT-PCR primers and probes.

Supplemental Table-S2. GO terms selectively enriched in Mesp1-CPCs.

Supplemental Table-S3. Echocardiographic characteristics of sham-operated controls and injected PBS or Mesp1-CPCs post-MI hearts.

Supplemental Figure-S1. Gene ontology networks negatively enriched in Mesp1-CPCs.

Supplemental Figure-S2. Establishment of the method to accurately detect Flk1, PDGFRa and Mesp1Cre-EYFP by FACS. Day 4 Mesp1Cre EYFP cells were stained by Flk1, PDGFRa, or both antibodies. The Flk1 antibody was detected by APC-conjugated secondary antibody, and the PDGFRa antibody was directly conjugated to PE. The none, single antibody, and double antibody labeling allowed accurate compensation of the three FACS detection channels (APC, PE and EYFP), so that each signal was only detected in its designated channel, without leaking into other detection channels.

Supplemental Figure-S3. Activin A does not cooperate with BMP4 in inducing/expanding the Mesp1Cre-EYFP+ population. Cells cultured as EBs were exposed to a serial of BMP4 (0.5 ng/ml, 1 ng/ml, 4 ng/ml and 16 ng/ml) and Activin A dosage (2 ng/ml, 4 ng/ml and 8 ng/ml). FACS was performed on day 4 cells. Increasing amount of BMP4 is correlated with more EYFP+ cells, while increasing amount of Activin A is not.

Supplemental Figure-S4. Supplemental Figure-S4. Cardiac function of injected MI+PBS and MI+Mesp1-CPCs hearts show the expected decreased in percent left ventricular fractional shortening and ejection fraction at 24-hours post-MI injection as measured by echocardiography. Echocardiographic parameters **(A)** Percent fractional shortening; LV-FS(%) and **(B)** Percent ejection fraction; LV-EF(%) of sham-operated controls and injected MI+PBS or MI+Mesp1-CPCs post-MI hearts. *Significance*: Data represented as the average +/- SEM, standard error of the mean; n = 5-6; 2-way ANOVA (Graph Pad, version 7). *Degree of significance*: *,†,§,¶ 0.1-0.05; **,††,§§,¶¶ 0.01-0.05; ***,†††,§§§,¶¶¶ 0.001-0.001; *Sequence of*

comparisons: * versus baseline; † versus 24-hours; § versus sham-operated control; ¶ versus MI+PBS

Supplemental Figure-S5. H&E serial sections of injected Mesp1-CPCs post-MI hearts show improved infarct repair at 6 weeks. (**A**) sham-operated control hearts, (**B**) MI hearts with intramyocardial injections of PBS control buffer and (**C**) MI hearts with intramyocardial injections of Mesp1-CPCs. Boxed area in (**C**) shows regions of cardiac repair of the infarct. *Scale bar* = 1 mm.

Supplemental Figure-S6. Masson trichrome staining of injected Mesp1-CPCs post-MI hearts show improved infarct repair at 12 weeks. (**A**) sham-operated control hearts; ***(a)*** magnified area in panel A, (**B**) MI hearts with intramyocardial injections of PBS control buffer (MI); ***(b)*** magnified area in panel B showing the forming scar in the free wall of the left ventricle (LV) in the PBS injected MI hearts (arrows), and (**C**) MI hearts with intramyocardial injections of Mesp1-CPCs (MI+cells); ***(c)*** magnified areas in panel C showing the reduced size of scar area of the free wall of the left ventricle (LV) in the CPC cells injected MI hearts, compared to B and b. (arrows). *Scale bar* = 1 μm.

Supplemental Figure-S7. Identification of Mesp1-EYFP+ cells in post-MI hearts. (**A**) EYFP+ myocardium was only detected in the border zone. A cross section of the infarct/border zone was stained by anti-YFP antibody, and panel B-E show a serial of magnified areas. (**B**, **C**) EYFP+ cells (arrow) in the border zone showed typical sarcomeric histology of cardiac myocytes. (**D**) Myocardium outside of the infarct/border zone was negative for anti-YFP staining. (**E**) EFYP+ endothelium-like structure in the border zone. (**F**, **G**) Magnified areas in panel E showing EYFP+ endothelium-like cells (arrow). (**H**) EYFP+ Endothelium-like cells in the infarct zone. *Scale bar* = 100 μm.

Supplemental Figure-S8. Border zone of MI+PBS in post-MI hearts show no GFP+/Ki67+ cells. Within the border zone of PBS injected post-MI hearts, no GFP+/Ki67+ cells were observed. The green round cells without nuclei are auto-fluorescent blood cells. (Note: *negative control for Figure-7H*). *Scale bar* = 100 μm.

Supplemental Table 1. Taqman realtime RT-PCR primers and probes

Gene	Forward primer	Probe	Reverse primer
Mesp1	caggaaaggcaggaaatgga	cagtccctcatctccgctcttcagca	ggtttctagaagagccagcatgtc
Nkx2-5	cctcgggcggataaaaaaga	cgcgctgcagaaggcagtgg	gccatccgtctcggctt
αMHC	gaatgacggacgcccagatg	ttgtcatcaggcacgaagcactccg	acgaccttggccttaacatactc
Ryr2	ctgttggcttattggctgttg	ccagcacggacgcccacatacat	ctgctgggtcttcaatttcatc
T	ctgctgggtcttcaatttcatc	taccccagcccctatgctcatcgg	tccgcataggttggagagct
Sox17	tcggtctggagagccatgag	tacgccagtgacgaccagagccagc	ccaccacctcgcctttcac
Flk1	actgcagtgattgccatgttct	ctggctccttcttgtcattgtcctacgga	tcattggcccgcttaacg
PDGFRa	ctggatgatctgcaagcatattaag	cctccacggtactcctgccacgg	cactttggcgaaggacactc
CDH5	caagatcagctcctccacgaag	cctgccattgctgccactgcca	gggtgtctatctggggaaagttag
Pecam1	cgaagttagagttctcctccagtc	tgtcactctcctcggcgatcttgct	gccgatgcctgcagtacag
Mef2c	tccactcccccattggact	accagaccttcgccggacgaaag	tgcgcttgactgaaggacttt
Tbx5	caggctgccttcacccag	agggcatggaaggaatcaaggtgtttct	cagccacagttcacgttcatg
Acta2	gccctgcctcatgccatc	cacggacaatctcacgctcggcagt	aagtccagagctacatagcacag

Supplemental Table 2. GO terms selectively enriched in Mesp1-CPCs

ID	Term	p value
GO:0048731	system development	1.62E-17
GO:0048856	anatomical structure development	1.91E-17
GO:0007275	multicellular organismal development	2.93E-16
GO:0048513	organ development	1.16E-14
GO:0032502	developmental process	2.43E-14
GO:0005515	protein binding	1.93E-12
GO:0032501	multicellular organismal process	8.24E-11
GO:0009653	anatomical structure morphogenesis	8.79E-11
GO:0007399	nervous system development	9.08E-10
GO:0007507	heart development	1.23E-09
GO:0030154	cell differentiation	1.68E-08
GO:0001944	vasculature development	2.28E-08
GO:0005488	binding	3.29E-08
GO:0048869	cellular developmental process	3.47E-08
GO:0001568	blood vessel development	4.01E-08
GO:0048519	negative regulation of biological process	4.29E-08
GO:0001701	in utero embryonic development	5.63E-08
GO:0048523	negative regulation of cellular process	7.61E-08
GO:0048522	positive regulation of cellular process	8.45E-08
GO:0009888	tissue development	9.74E-08
GO:0009887	organ morphogenesis	1.36E-07
GO:0048518	positive regulation of biological process	1.67E-07
GO:0009790	embryonic development	2.29E-07
GO:0043009	chordate embryonic development	7.58E-07
GO:0009792	embryonic development ending in birth or egg hatching	1.01E-06
GO:0001501	skeletal system development	1.23E-06
GO:0042127	regulation of cell proliferation	4.62E-06
GO:0043231	intracellular membrane-bounded organelle	6.07E-06
GO:0061061	muscle structure development	6.98E-06
GO:0043227	membrane-bounded organelle	7.04E-06
GO:0009892	negative regulation of metabolic process	8.42E-06
GO:0031323	regulation of cellular metabolic process	8.69E-06
GO:0016564	transcription repressor activity	8.73E-06
GO:0019222	regulation of metabolic process	8.81E-06

Supplemental Table-3. Echocardiographic characteristics of sham-operated controls and injected PBS or Mesp1-CPCs post-MI hearts.

Study Group	Echo Parameter	Baseline	24-hours	7-days	14-days
**Sham**		0.92 ± 0.05	1.11 ± 0.11 NS	1.12 ± 0.08 NS	1.06 ± 0.07 NS
**MI+PBS**	**LV-AW;d (mm)**	1.03 ± 0.06	1.04 ± 0.06 NS	1.16 ± 0.06 NS	1.08 ± 0.10 NS
**MI+CPC**		0.95 ± 0.07	0.98 ± 0.12 NS	0.92 ± 0.09 NS	1.02 ± 0.08 NS
**Sham**		1.28 ± 0.03	1.45 ± 0.10 NS	1.53 ± 0.06 NS	1.51 ± 0.04 NS
**MI+PBS**	**LV-AW;s (mm)**	1.43 ± 0.09	1.28 ± 0.06 NS	1.49 ± 0.12 NS	1.30 ± 0.13 NS
**MI+CPC**		1.26 ± 0.07	1.15 ± 0.11 NS	1.25 ± 0.14 NS	1.31 ± 0.12 NS
**Sham**		0.81 ± 0.08	0.81 ± 0.03 NS	1.08 ± 0.08 NS	0.91 ± 0.04 NS
**MI+PBS**	**LV-PW;d (mm)**	0.80 ± 0.06	1.17 ± 0.17 **§§	1.16 ± 0.14 **	1.15 ± 0.06 *
**MI+CPC**		0.99 ± 0.07	0.91 ± 0.08 NS	1.16 ± 0.13 NS	1.03 ± 0.09 NS
**Sham**		1.08 ± 0.08	1.13 ± 0.07 NS	1.30 ± 0.06 NS	1.27 ± 0.05 NS
**MI+PBS**	**LV-PW;s (mm)**	1.15 ± 0.09	1.39 ± 0.20 NS	1.58 ± 0.18 **	1.56 ± 0.10 **§§
**MI+CPC**		1.29 ± 0.05	1.18 ± 0.11 NS	1.47 ± 0.10 NS	1.40 ± 0.09 ¶¶¶
**Sham**		3.69 ± 0.09	3.71 ± 0.08 NS	3.54 ± 0.15 NS	3.69 ± 0.08 NS
**MI+PBS**	**LV-ID;d (mm)**	3.82 ± 0.08	3.77 ± 0.11 NS	3.78 ± 0.13 NS	4.25 ± 0.33 †§§
**MI+CPC**		3.49 ± 0.04	3.71 ± 0.11 NS	3.54 ± 0.16 NS	3.50 ± 0.18 ¶¶¶
**Sham**		2.70 ± 0.05	2.61 ± 0.13 NS	2.52 ± 0.07 NS	2.55 ± 0.08 NS
**MI+PBS**	**LV-ID;s (mm)**	2.65 ± 0.15	2.97 ± 0.14 NS	2.74 ± 0.22 NS	3.29 ± 0.36 NS
**MI+CPC**		2.47 ± 0.11	2.99 ± 0.13 NS	2.59 ± 0.23 NS	2.41 ± 0.28 NS
**Sham**		26.83 ± 0.68	29.66 ± 3.26 NS	28.60 ± 1.53 NS	31.03 ± 1.32 NS
**MI+PBS**	**LV-FS (%)**	30.90 ± 2.99	21.23 ± 1.78 *§	28.07 ± 3.62 NS	23.59 ± 3.32 NS
**MI+CPC**		29.00 ± 3.33	19.38 ± 2.68 *§§	27.26 ± 3.23 NS	32.01 ± 4.20 ††¶
**Sham**		53.16 ± 1.04	56.90 ± 4.91 NS	55.94 ± 2.33 NS	59.46 ± 2.00 NS
**MI+PBS**	**LV-EF (%)**	58.61 ± 4.19	43.60 ± 3.14 *§	50.38 ± 4.92 NS	46.77 ± 5.64 §
**MI+CPC**		55.91 ± 5.21	40.43 ± 2.95 *§§	53.40 ± 5.58 NS	60.18 ± 6.88 ††¶

- LV-AW, left ventricular anterior wall thickness; LVP-D, left ventricular posterior wall thickness; LV-ID, left ventricular internal diameter LV-FS, left ventricular fractional shortening; LV, left ventricular fractional shortening; LV-EF, left ventricular ejection fration; d, diastolic; s, systolic

- Equations: LV-FS (%) = [(LV-ID(d) – LV-ID(s)) / LV-ID(d)] x 100; LV-EF (%) = [(LV-Vol;d – LV-Vol;s) / LV-Vol;d] x 100; where LV chamber volume is derived from the Teichholz formula: LV-Vol;d, left ventricular volume diastole (ul) = ((7.0 / (2.4 + LV-ID(d))) * LV-ID(d)^3 LV-Vol(s), left ventricular volume systole (ul) = ((7.0 / (2.4 + LV-ID(s))) * LV-ID(s)^3

- Significance: Data represented as the average +/- SEM, standard error of the mean; n = 5-6; 2-way ANOVA (GraphPad, version 7)
  Degree of significance: *,†,§,¶ 0.1-0.05; **,††,§§,¶¶ 0.01-0.05; ***,†††,§§§,¶¶¶ 0.001-0.001
  Sequence of comparisions: * versus baseline; † versus 24-hours; § versus sham-operated control; ¶ versus MI+PBS

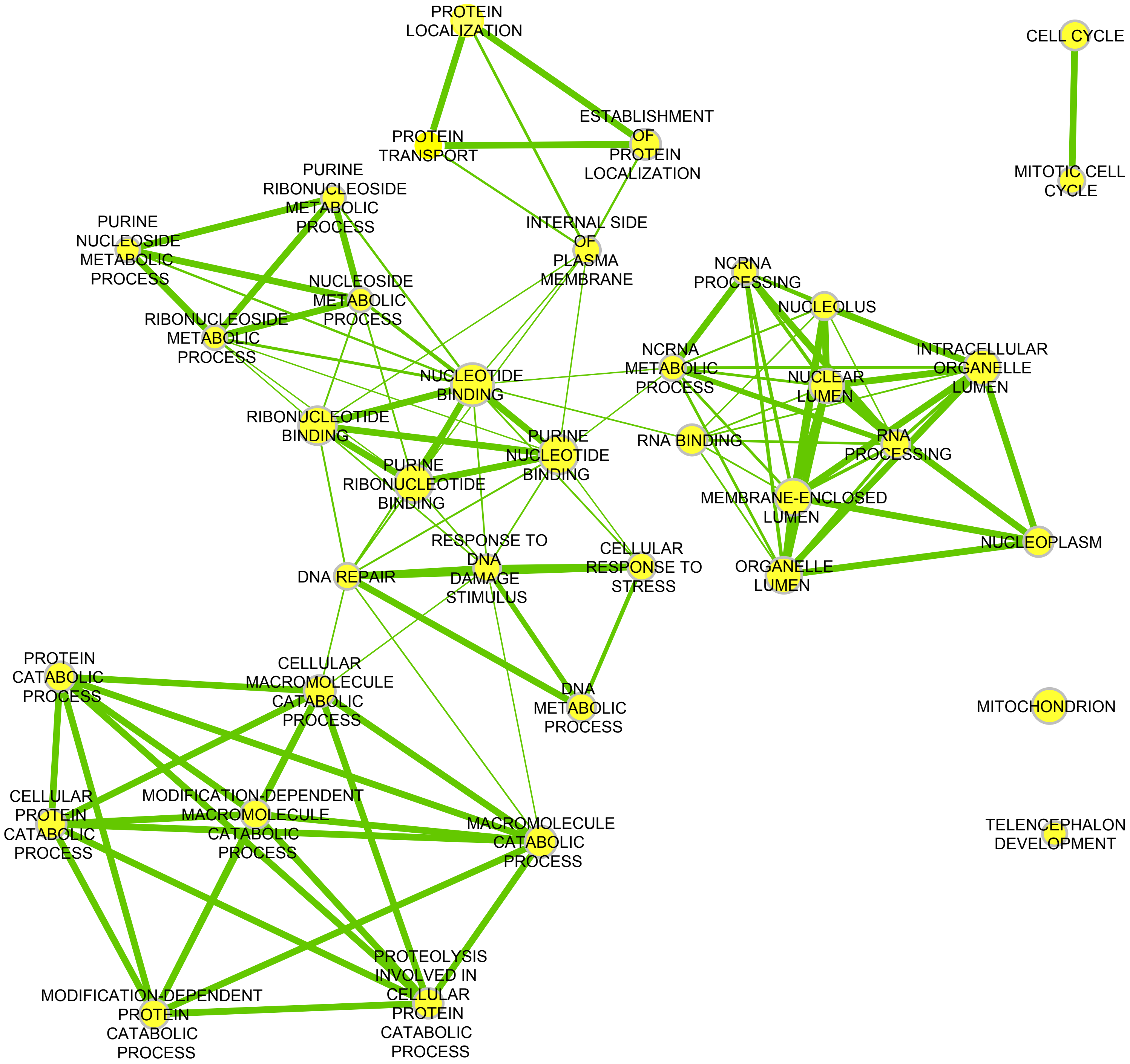

Supplemental Figure-S1. Gene ontology networks negatively enriched in Mesp1-CPCs.

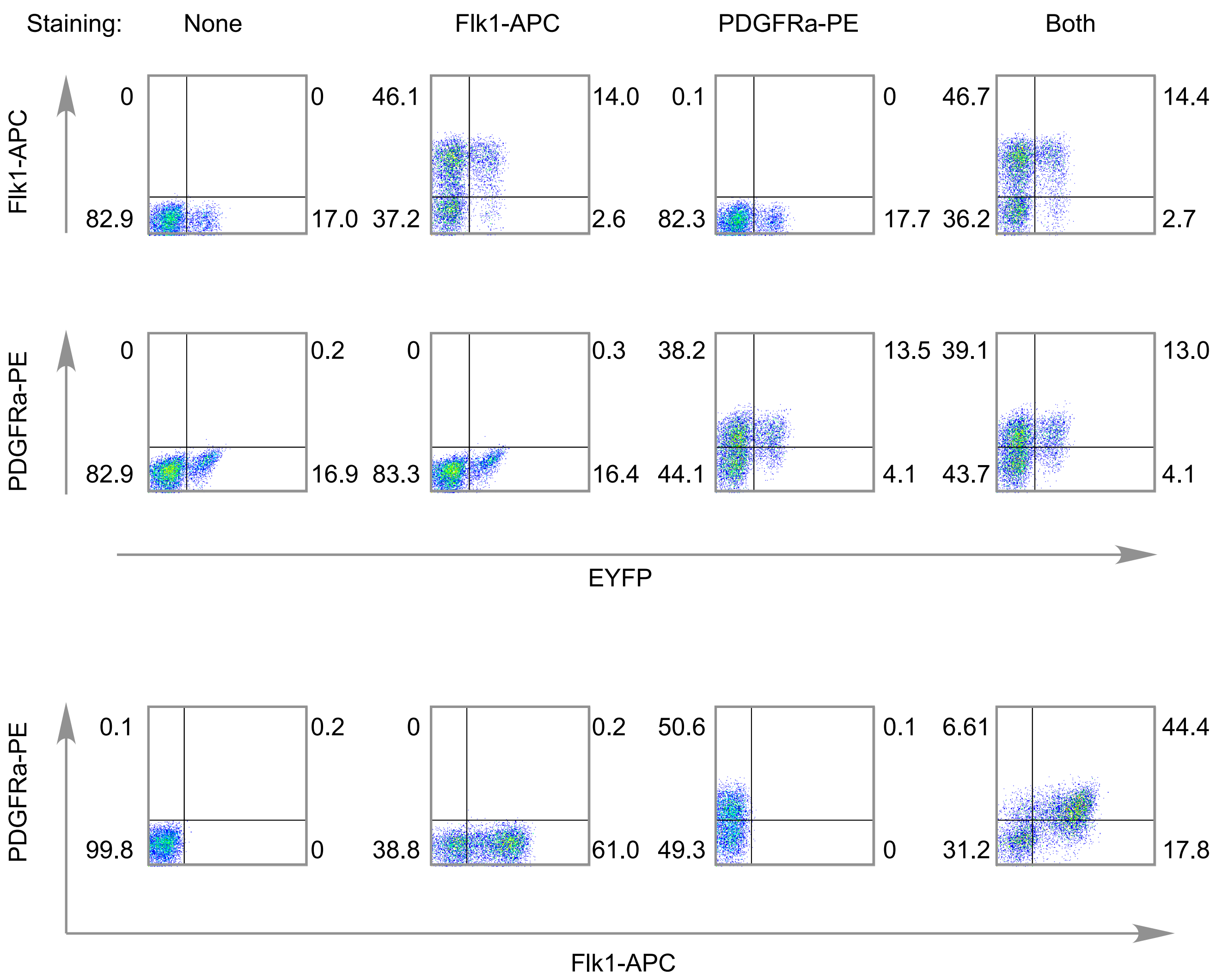

Supplemental Figure-S2. Establishment of the method to accurately detect Flk1, PDGFRa and Mesp1-EYFP+ cells by FACS.

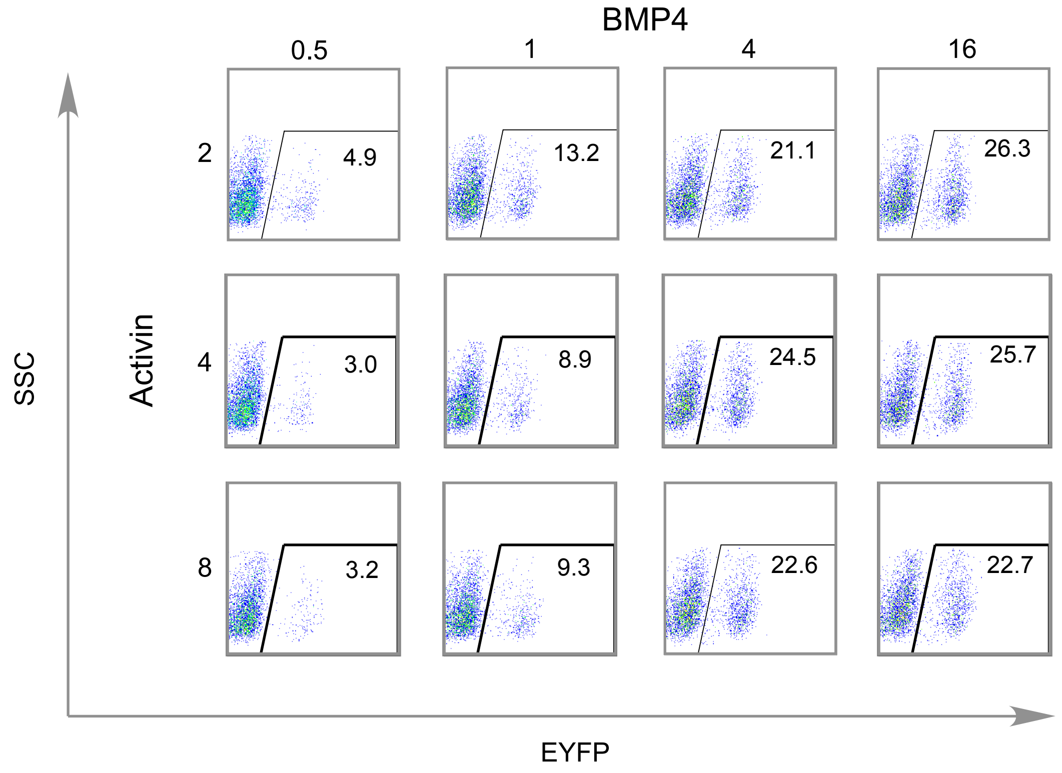

Supplemental Figure-S3. Activin A did not cooperate with BMP4 in inducing/expanding Mesp1-CPCs.

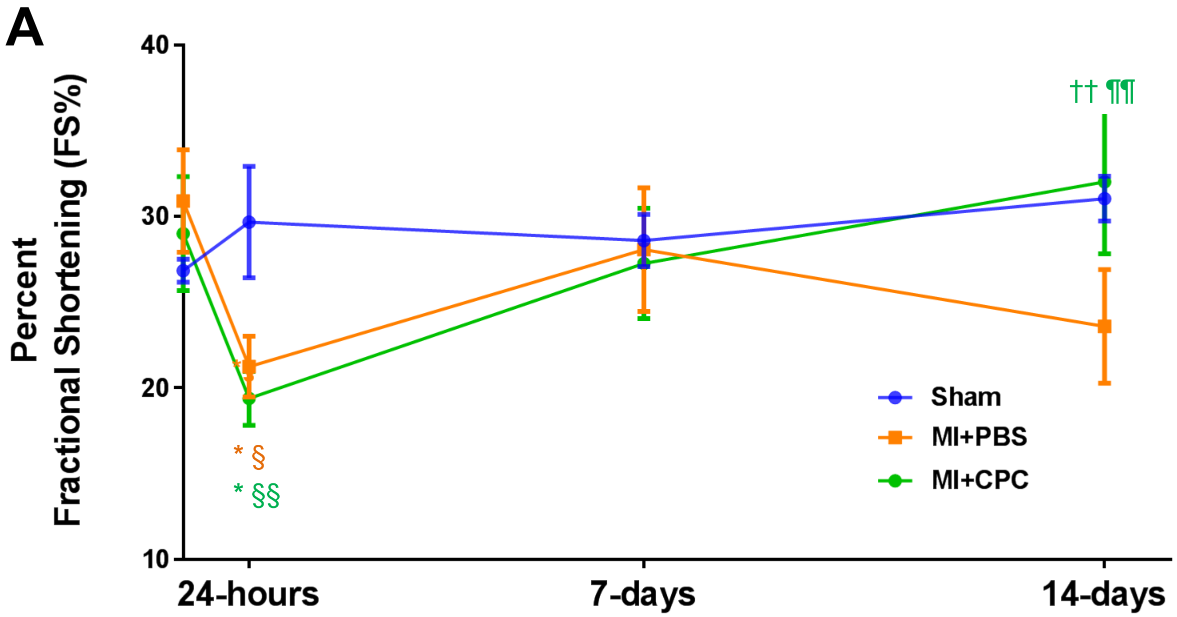

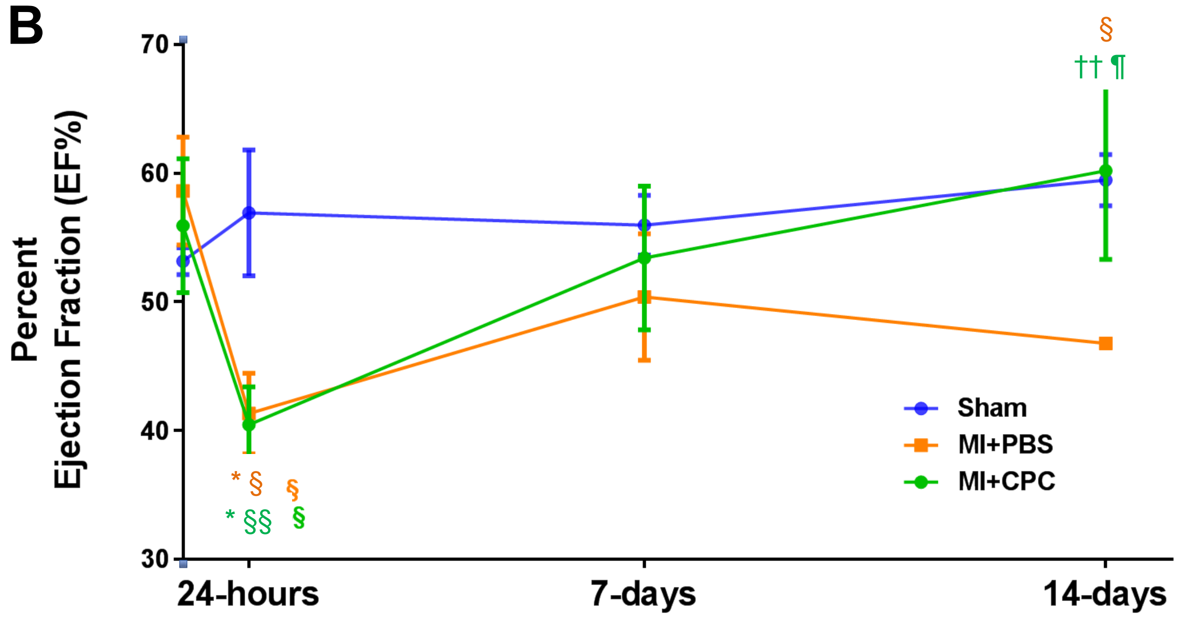

Supplemental Figure-S4. Cardiac function of injected MI+PBS and MI+Mesp1-CPCs hearts show the expected decreased in percent left ventricular fractional shortening and ejection fraction at 24-hours post-MI injection as measured by echocardiography.

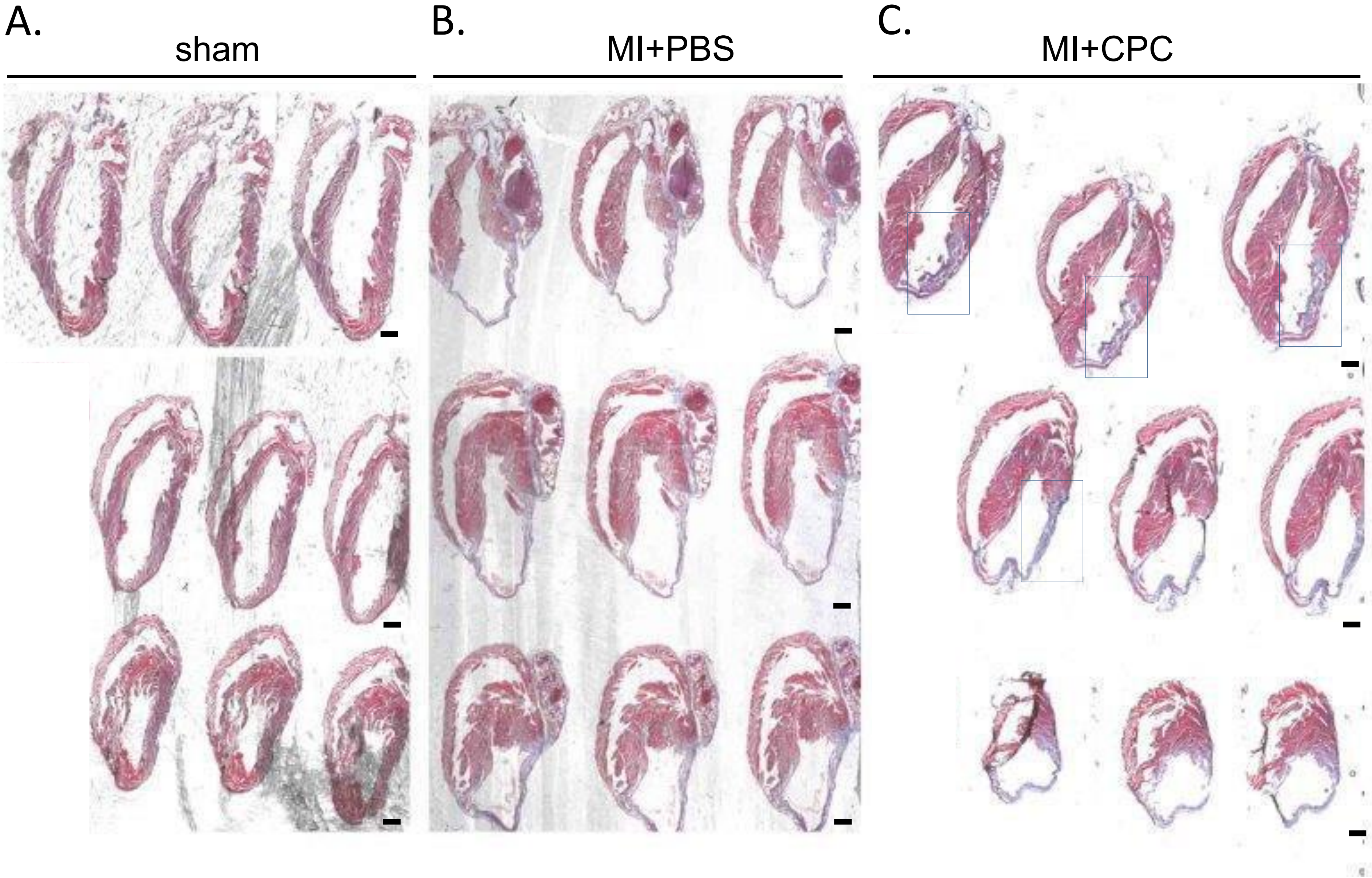

Supplemental Figure-S5. H&E serial sections of injected Mesp1-CPCs post-MI hearts show improved infarct repair (*Scale bar:1mm*).

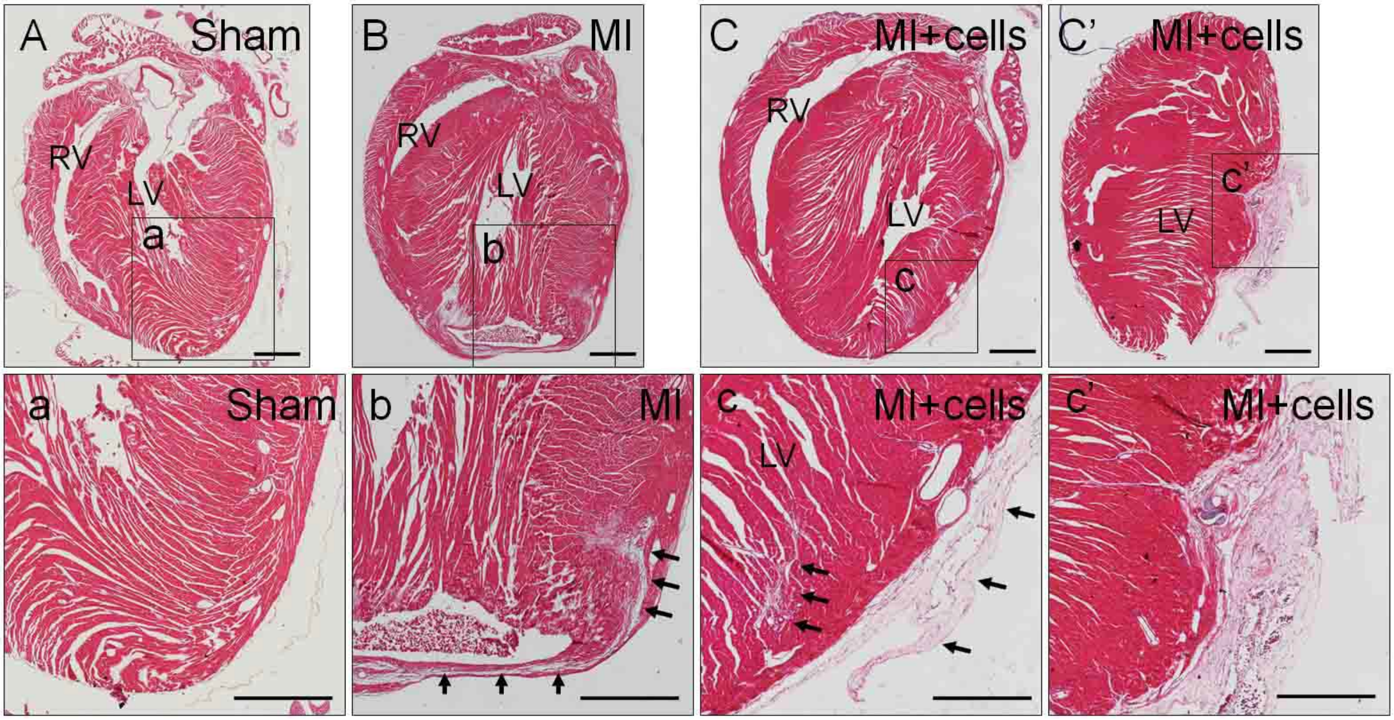

Supplemental Figure-S6. Masson trichrome staining of injected Mesp1-CPCs post-MI hearts show improved infarct repair at 12 weeks (MI = MI+PBS; MI+cells = MI+CPC; and *Scale bar: 1μm*).

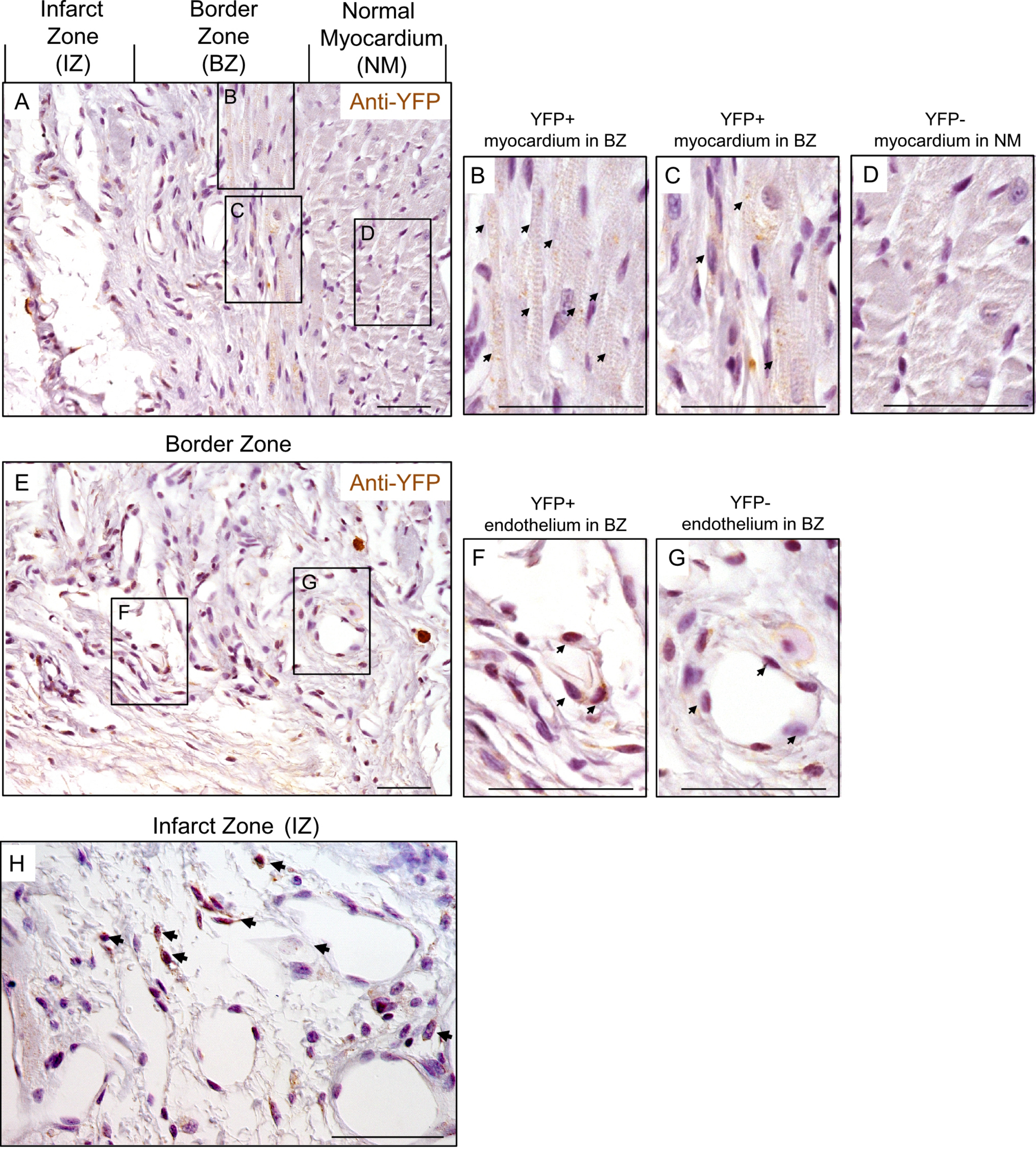

Supplemental Figure-S7. Identification of Mesp1-CPCs (EYFP+) cells in post-MI hearts (*Scale bar: 100um*).

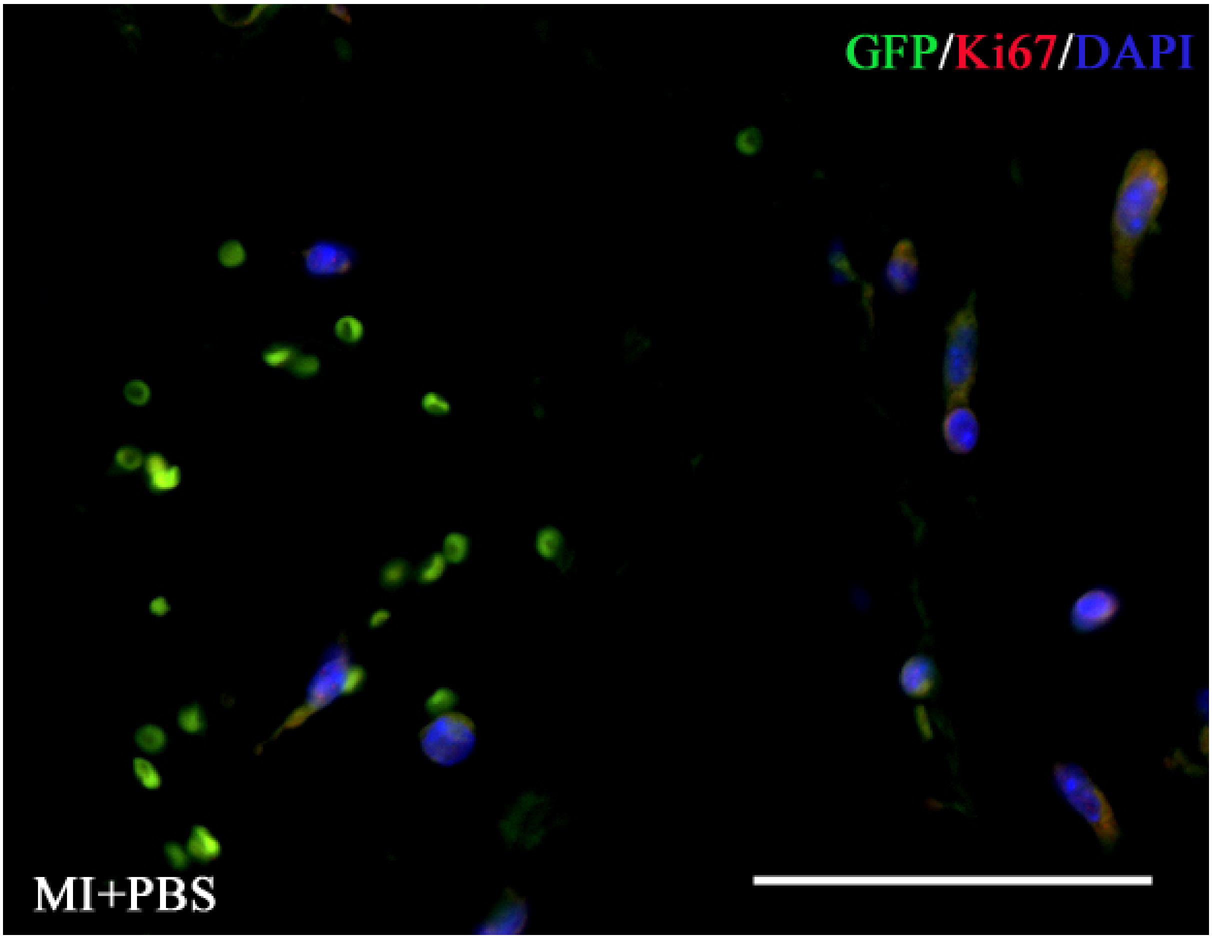

Supplemental Figure-S8. Border zone of MI+PBS in post-MI hearts show no GFP+/Ki67+ cells (*negative control for Figure-7H*).
