# Supplementary figures and images for: Mesp1 Marked Cardiac Progenitor Cells Repair Infarcted Mouse Hearts
Source: Sci Rep. 2016 Aug 19;6:31457. doi: 10.1038/srep31457 (PMC4990963; doi:10.1038/srep31457)

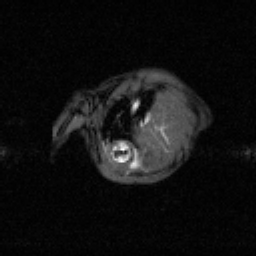

Supplement: Supplementary Movie S1 [file srep31457-s2.gif]

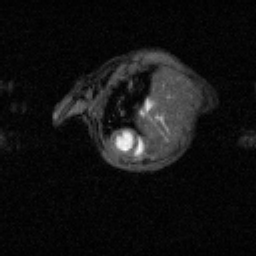

Supplement: Supplementary Movie S2 [file srep31457-s3.gif]

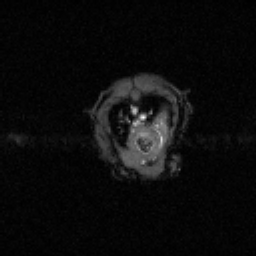

Supplement: Supplementary Movie S3 [file srep31457-s4.gif]

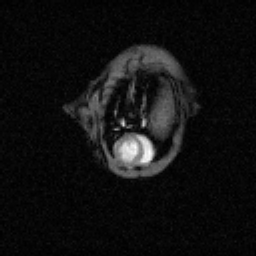

Supplement: Supplementary Movie S4 [file srep31457-s5.gif]

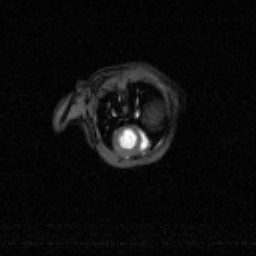

Supplement: Supplementary Movie S5 [file srep31457-s6.gif]

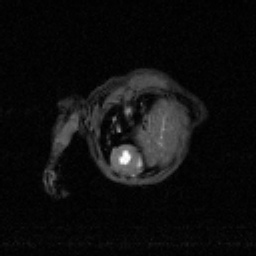

Supplement: Supplementary Movie S6 [file srep31457-s7.gif]
